# Supplementary material for: The tumor microenvironment of 14,837 breast cancers is associated with clinical outcome independently of genomic subtypes
Source: Cell Rep Med. 2025 Nov 10;6(11):102450. doi: 10.1016/j.xcrm.2025.102450 (PMC12711693; doi:10.1016/j.xcrm.2025.102450)
Supplement: Document S1. Figures S1–S10 [file mmc1.pdf]

Cell Reports Medicine, Volume *6*

Supplemental information

The tumor microenvironment of 14,837 breast cancers is associated with clinical outcome independently of genomic subtypes

Kevin J. Tu, Daniel Guerrero-Romero, Kate Eason, Raquel Manzano Garcia, Jia Wern, Soo-Hwang Teo, Long Nguyen, Stephen-John Sammut, Florian Markowetz, Oscar M. Rueda, and Carlos Caldas

Supplementary Figures

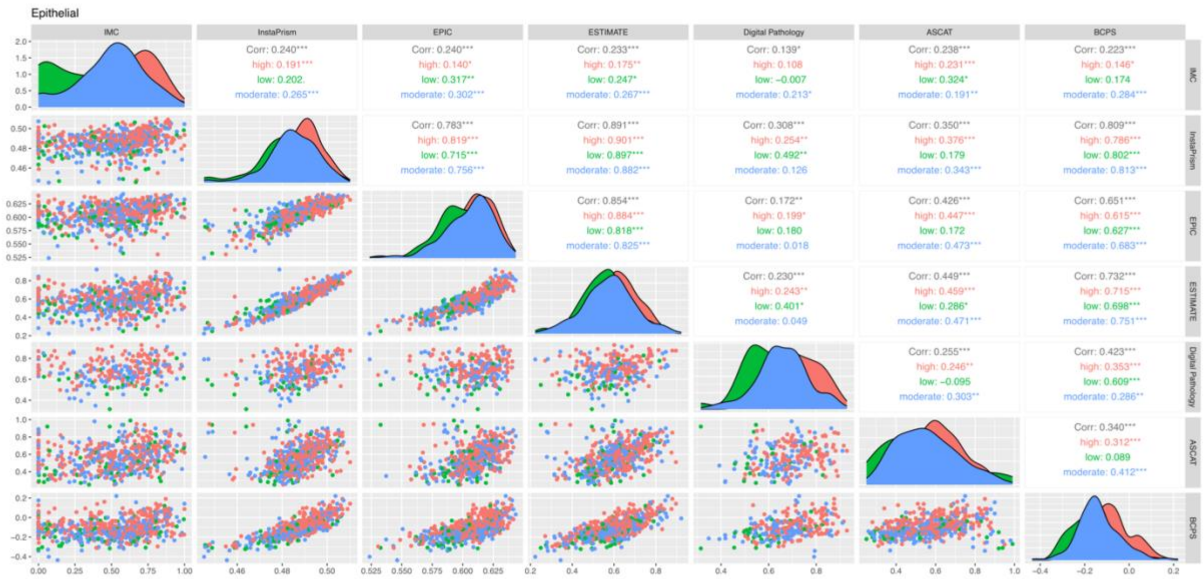

Supplementary Figure 1. Scatterplot matrix of epithelial characterization. Red is high cellularity samples, green is low cellularity samples, and blue is moderate cellularity samples according to pathologist's assessment. * p < 0.05, ** p < 0.01, *** p < 0.001, **** p < 0.0001. Related to Figure 1.

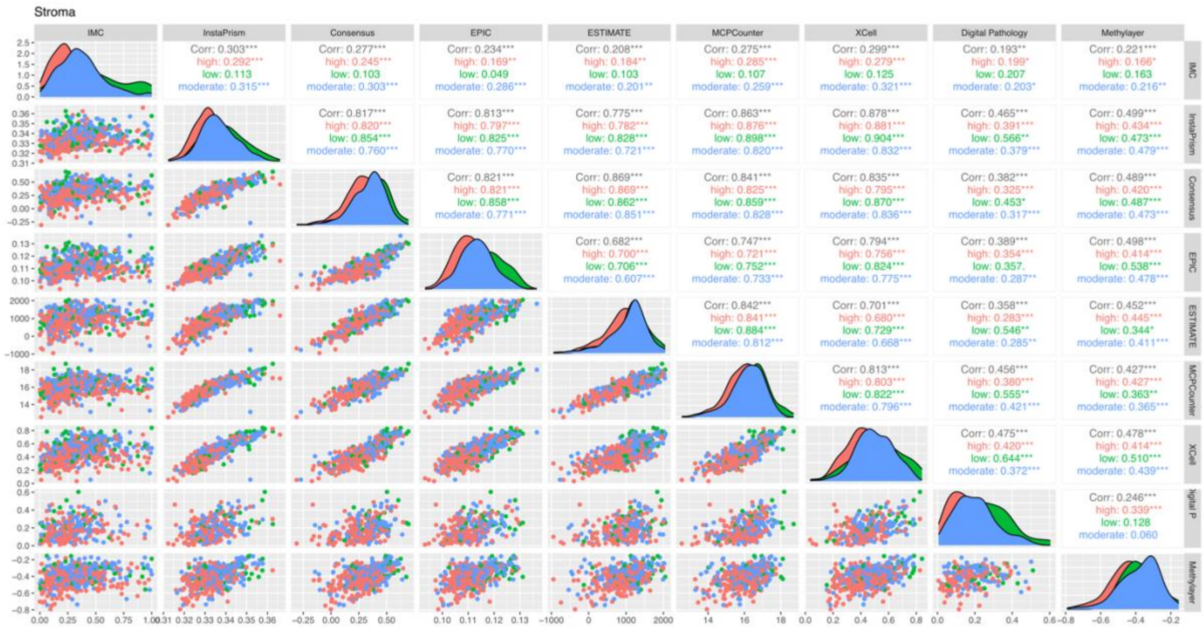

Supplementary Figure 2. Scatterplot matrix of stroma characterization. Red is high cellularity samples, green is low cellularity samples, and blue is moderate cellularity samples. * p < 0.05, ** p < 0.01, *** p < 0.001, **** p < 0.0001. Related to Figure 1.

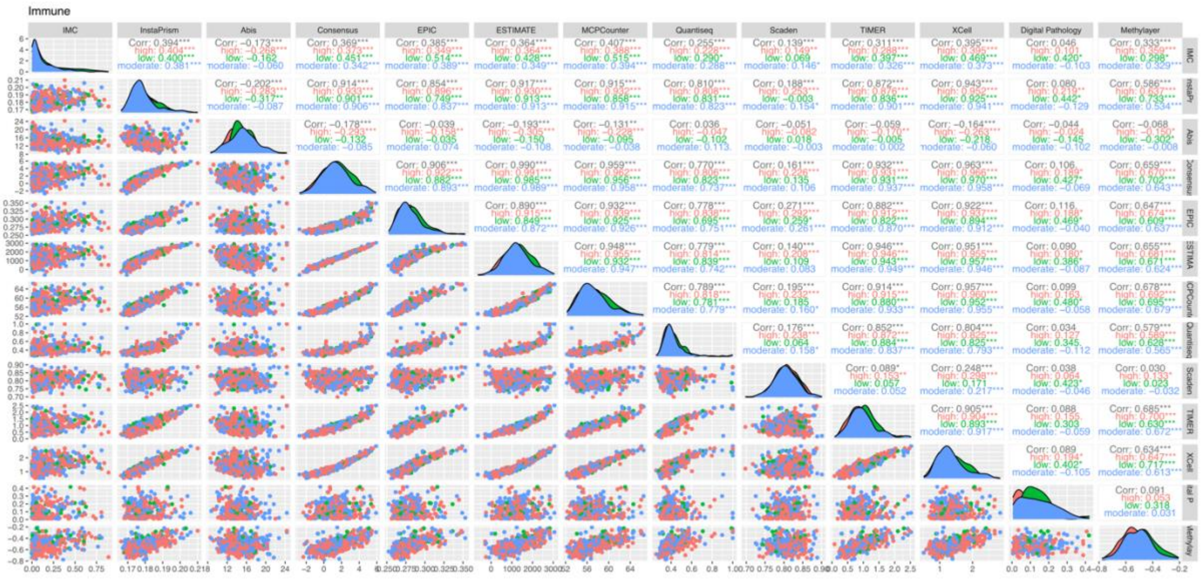

Supplementary Figure 3. Scatterplot matrix of immune characterization. Red is high cellularity samples, green is low cellularity samples, and blue is moderate cellularity samples. * $p < 0.05$, ** $p < 0.01$, *** $p < 0.001$, **** $p < 0.0001$. Related to Figure 1.

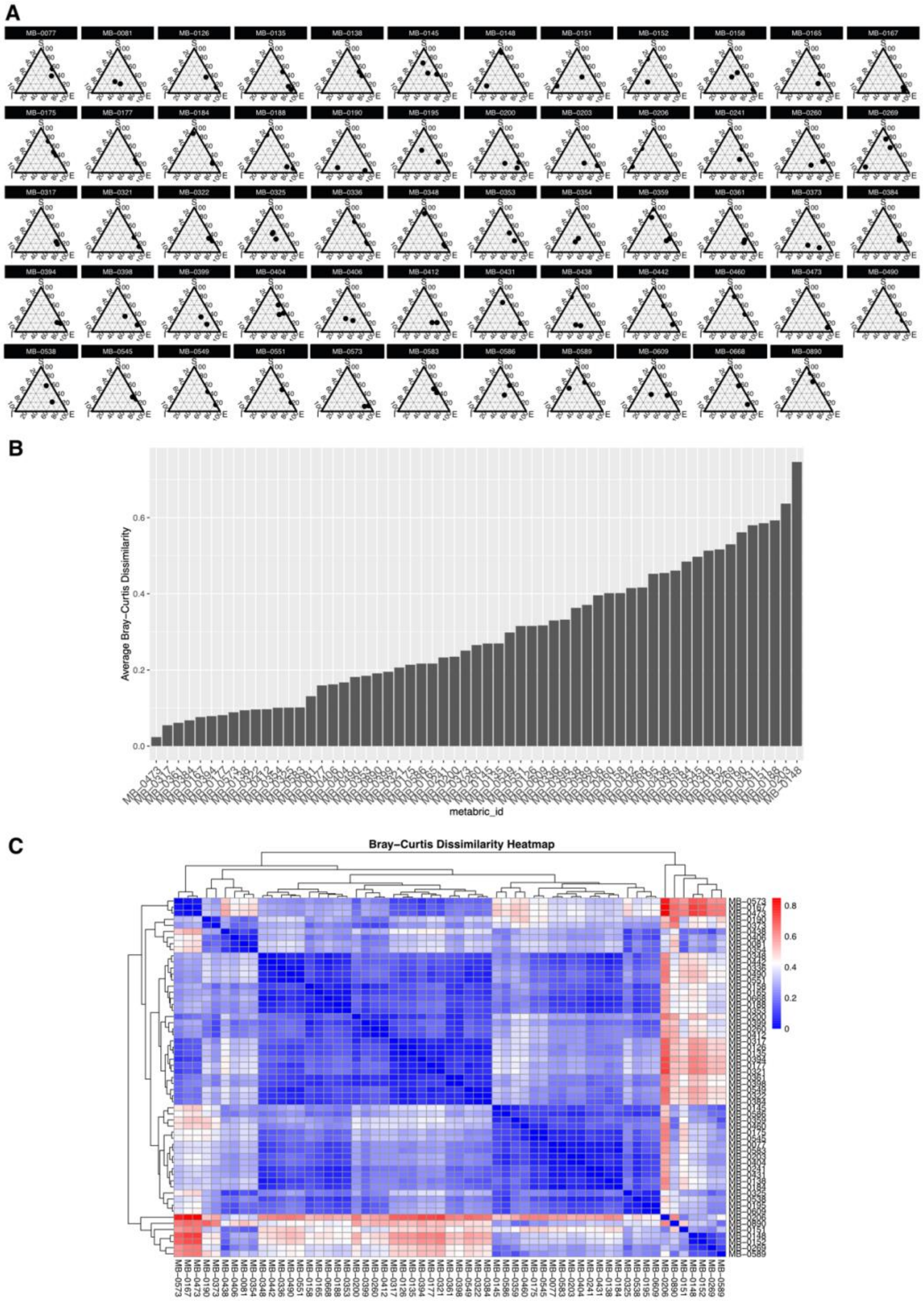

Supplementary Figure 4. IMC Sample Variation. A) Ternary plots representing the stroma (S), immune (I), and epithelial (E) fractions of patients with multiple IMC samples. B) Average pairwise Bray-Curtis dissimilarity between sample makeup for each tumor. C) Dissimilarity matrix of the average pairwise Bray-Curtis dissimilarity between tumors, clustering by Euclidean distance. Related to Figure 1.

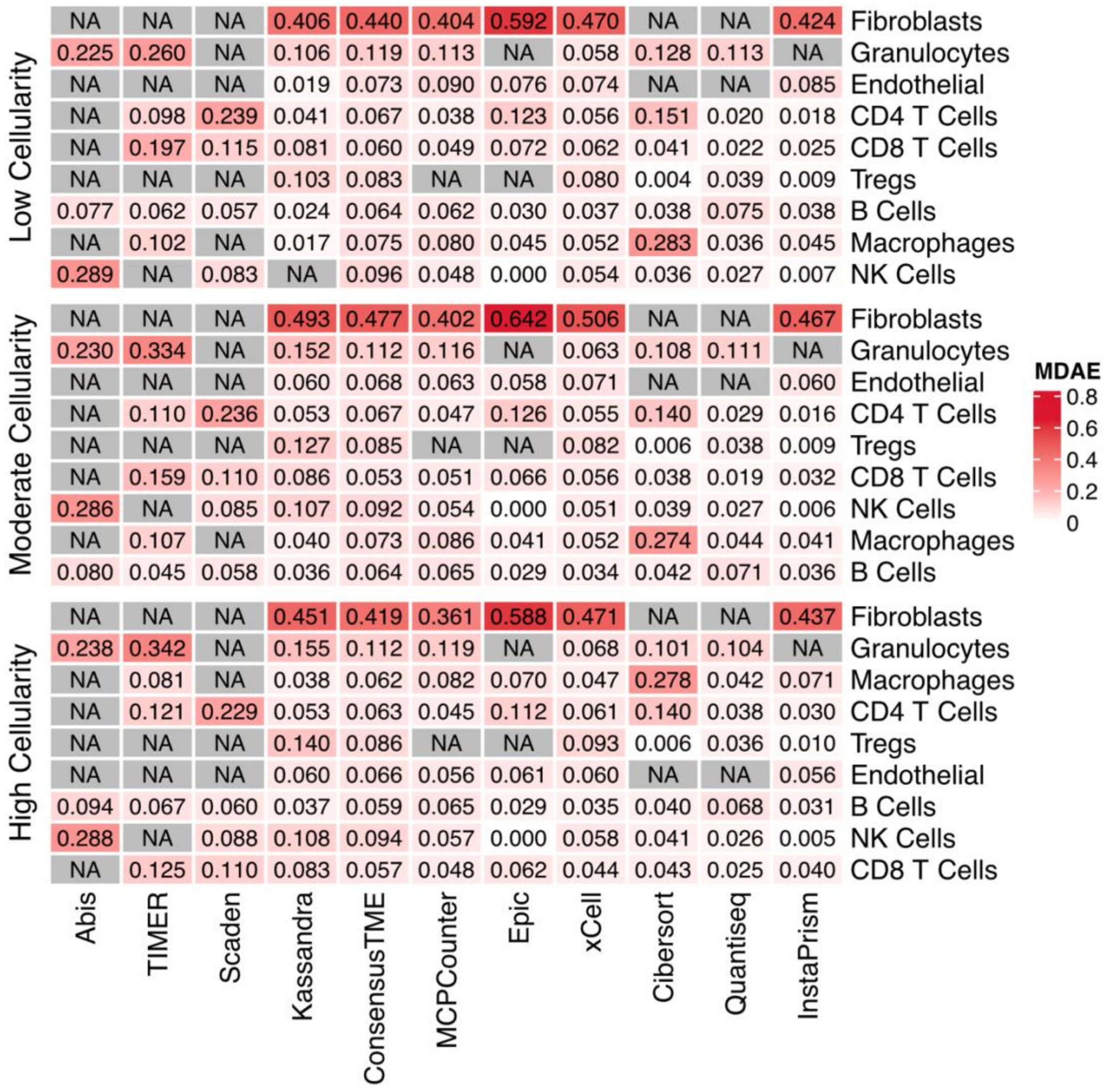

Supplementary Figure 5. Deconvolution algorithm benchmarking using MDAE. Samples are ordered from left to right and top to bottom by decreasing median MDAE. Related to Figure 1.

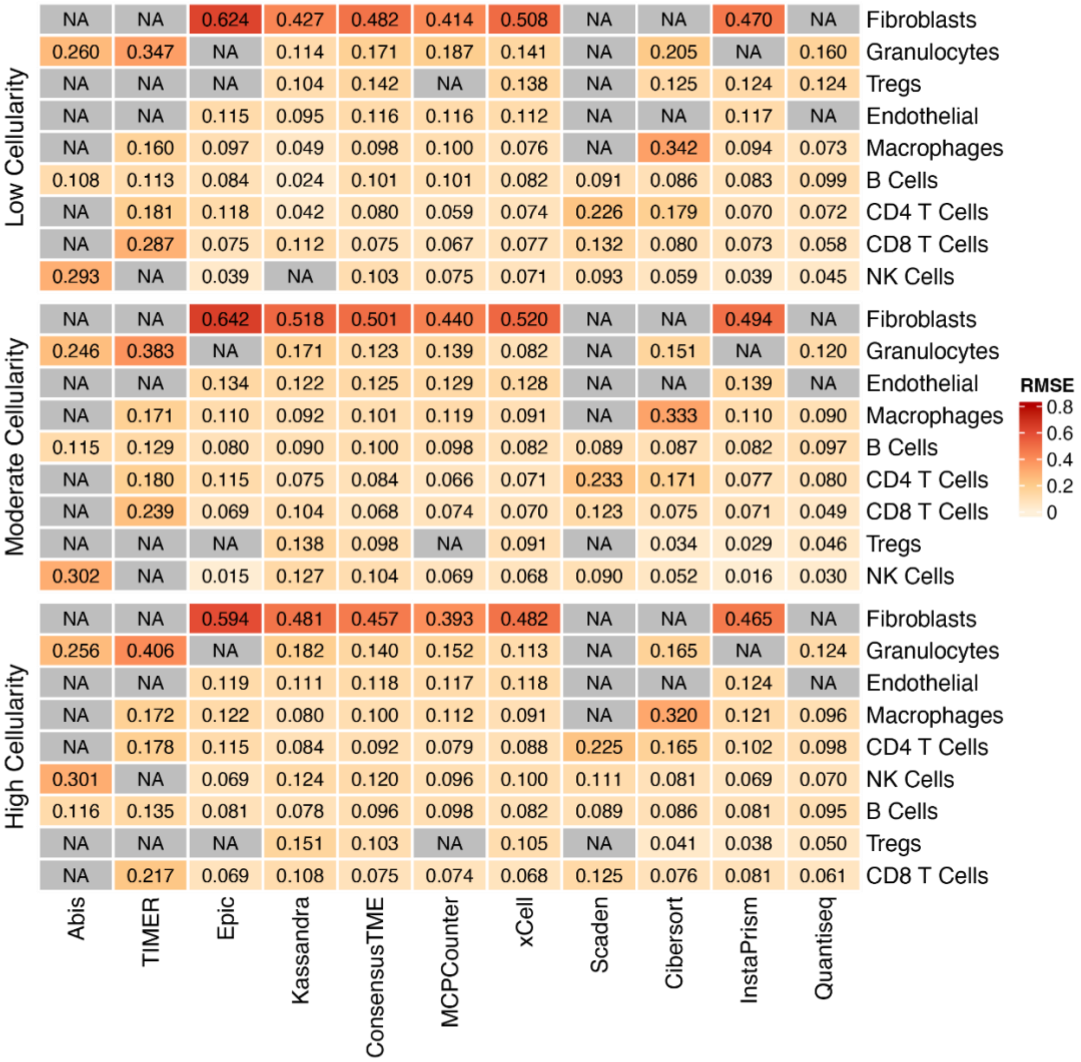

Supplementary Figure 6. Deconvolution algorithm benchmarking using RMSE. Samples are ordered from left to right and top to bottom by decreasing median RMSE. Related to Figure 1.

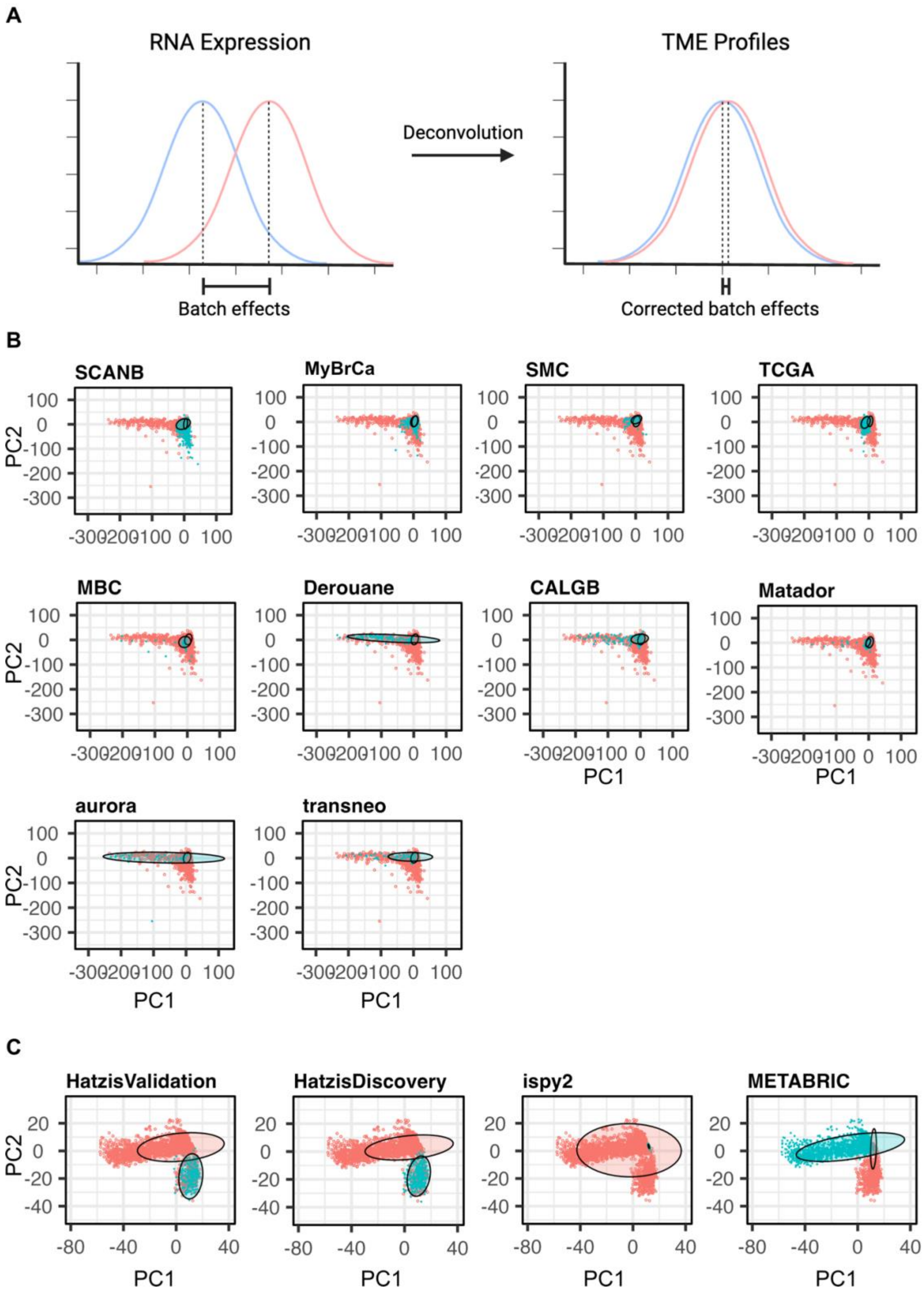

Supplementary Figure 7. Correction of batch effects by deconvolution.

A) Fictional example of translation of TME proportions to account for batch effects through deconvolution. B) PCA plots colored by RNAseq study, the study is in blue and all other studies are in red. C) PCA plots colored by microarray study, the study is in blue and all other studies are in red. Related to Figure 2.

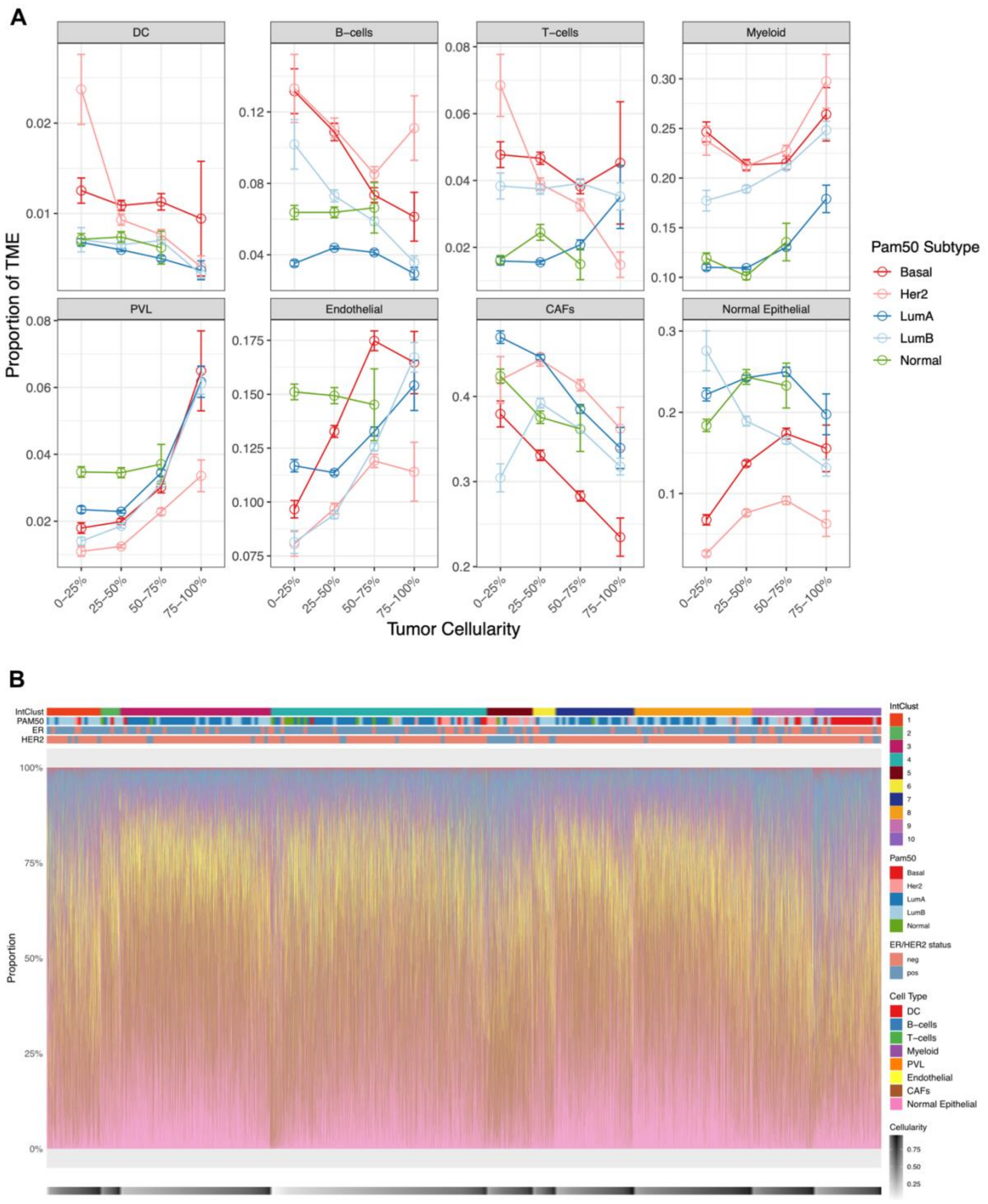

Supplementary Figure 8. TME profiles in Pam50 and IntClust subtypes. A) Comparing cell types between the instrinsic subtypes over cellularity. B) The TME profiles of all patients in our meta-dataset grouped by integrative cluster. Related to Figure 3.

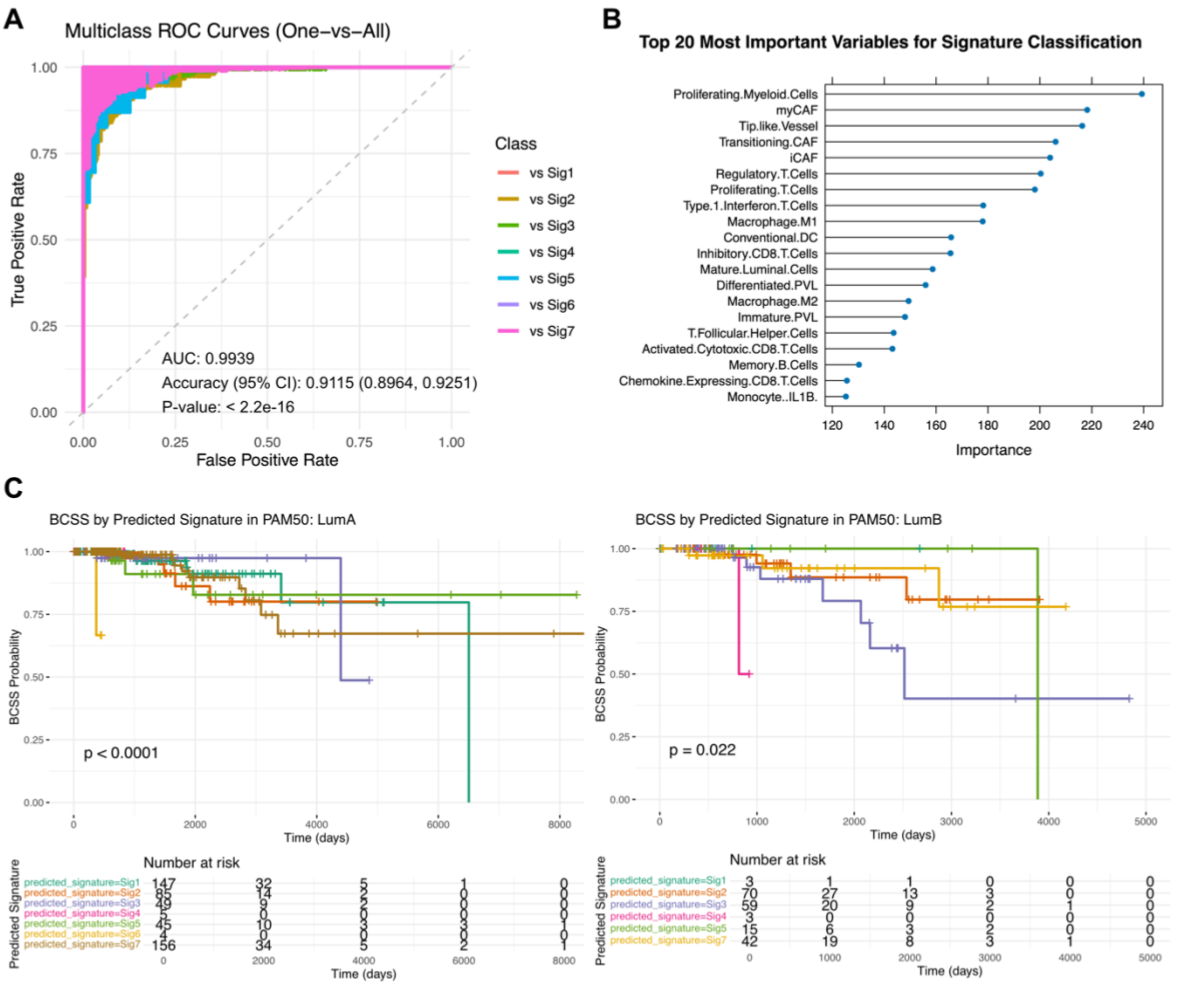

Supplementary Figure 9. Validation of Ecotypes within the TCGA cohort. A) AUC curve of random forest classifier. B) Confusion matrix of random forest classifier. C) Variable importance for the classifier. D) BCSS KM curves of TME types within Luminal subtypes. Related to Figure 4.

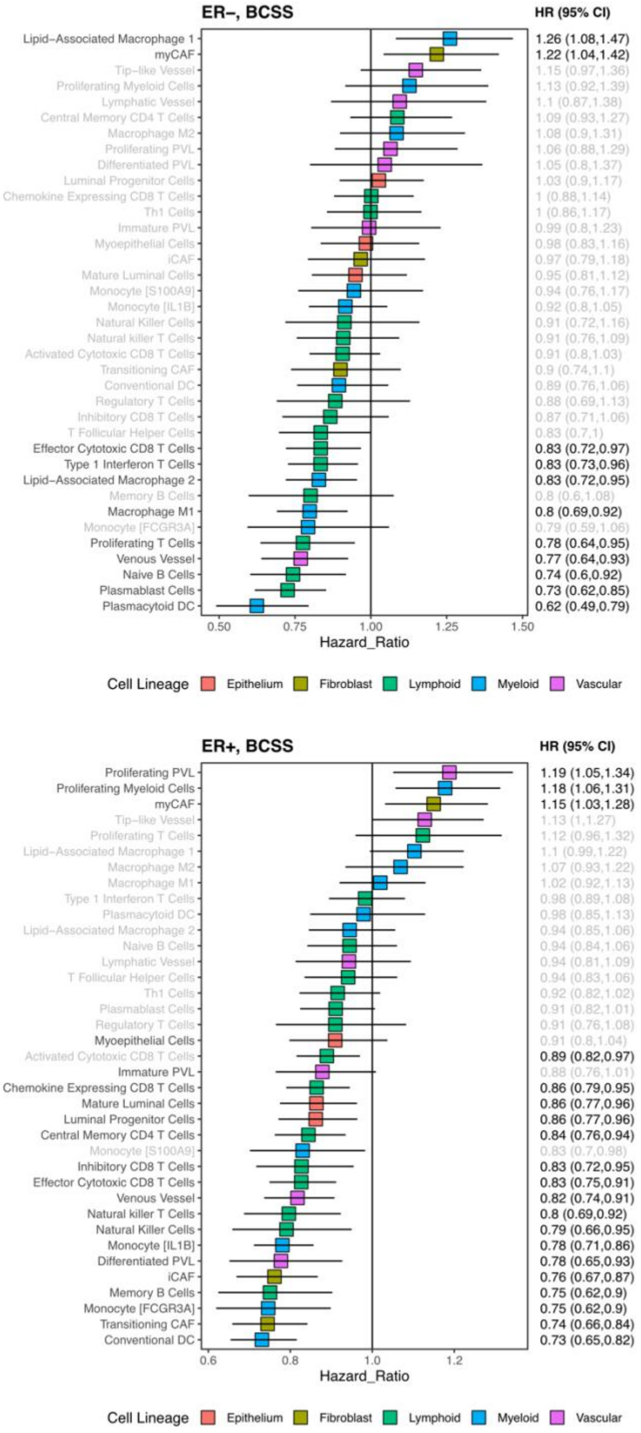

Supplementary Figure 10. The effects of TME features on disease-specific survival. A) Adjusted Cox model in ER+ patients for BCSS. B) Adjusted Cox model in ER- patients for BCSS. Related to Figure 5.
