# Supplementary material for: The tumor microenvironment of 14,837 breast cancers is associated with clinical outcome independently of genomic subtypes
Source: Cell Rep Med. 2025 Nov 10;6(11):102450. doi: 10.1016/j.xcrm.2025.102450 (PMC12711693; doi:10.1016/j.xcrm.2025.102450)
Supplement: Document S2. Article plus supplemental information [file mmc8.pdf]

Cell Reports
Medicine

Article

The tumor microenvironment of 14,837 breast cancers is associated with clinical outcome independently of genomic subtypes

Graphical abstract

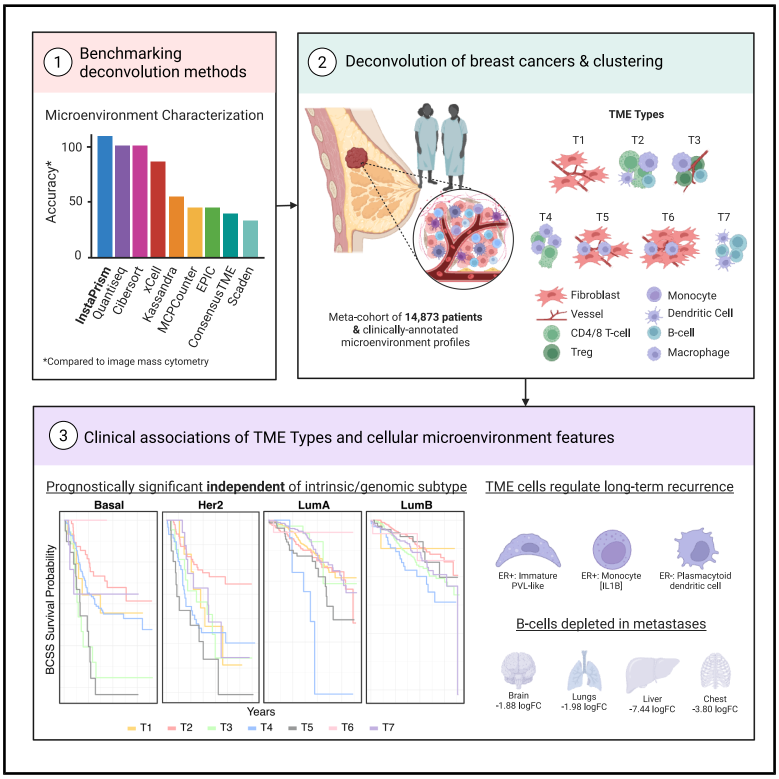

Highlights

- 15 microenvironment deconvolution methods benchmarked across 693 breast cancers
- Deconvolution and clustering of 14,837 breast cancers identify 7 “TME types”
- TME types are prognostically distinct, independent of intrinsic and genomic subtypes
- Microenvironment-tumor interactions shape chemo response, recurrence, and metastases

Authors

Kevin J. Tu, Daniel Guerrero-Romero, Kate Eason, ..., Florian Markowetz, Oscar M. Rueda, Carlos Caldas

Correspondence

kevin.tu@som.umaryland.edu (K.J.T.),
oscar.rueda@mrc-bsu.cam.ac.uk (O.M.R.),
cc234@cam.ac.uk (C.C.)

In brief

Tu et al. present a systematic analysis of the tumor microenvironment across 14,837 breast cancers, identifying distinct cellular patterns that predict disease-specific survival independently of genomic and intrinsic subtypes. Characterizing microenvironment composition may provide critical prognostic markers that can complement current molecular classification for treatment selection and preventing recurrence.

Tu et al., 2025, Cell Reports Medicine *6*, 102450
November 18, 2025 © 2025 The Author(s). Published by Elsevier Inc.
https://doi.org/10.1016/j.xcrm.2025.102450

CellPress

Cell Reports Medicine

CellPress
OPEN ACCESS

Article

The tumor microenvironment of 14,837 breast cancers is associated with clinical outcome independently of genomic subtypes

Kevin J. Tu,[1,2,11,*] Daniel Guerrero-Romero,[1] Kate Eason,[1] Raquel Manzano Garcia,[1] Jia Wern,[3] Soo-Hwang Teo,[3,4] Long Nguyen,[5] Stephen-John Sammut,[6,7] Florian Markowetz,[1] Oscar M. Rueda,[8,*] and Carlos Caldas[9,10,*]
[1]Cancer Research UK Cambridge Institute, University of Cambridge, Cambridge, UK
[2]University of Maryland School of Medicine, Baltimore, MD, USA
[3]Cancer Research Malaysia, No. 1, Jalan SS12/1A, Subang Jaya 47500, Malaysia
[4]University Malaya Cancer Research Institute, Faculty of Medicine, University Malaya, Kuala Lumpur, Malaysia
[5]Department of Medicine, University of Toronto, Toronto, ON, Canada
[6]Breast Cancer Now Toby Robins Research Centre, Institute of Cancer Research, London, UK
[7]The Royal Marsden Hospital NHS Foundation Trust, London, UK
[8]MRC Biostatistics Unit, University of Cambridge, East Forvie Building, Forvie Site, Robinson Way, Cambridge Biomedical Campus, CB2 0SR Cambridge, UK
[9]Institute of Metabolic Science, Department of Clinical Biochemistry, University of Cambridge, Cambridge, UK
[10]The Concern Foundation Laboratories at the Lautenberg Center for Immunology and Cancer Research, Institute for Medical Research Israel-Canada, Faculty of Medicine, Hebrew University of Jerusalem, Jerusalem, Israel
[11]Lead contact
*Correspondence: kevin.tu@som.umaryland.edu (K.J.T.), oscar.rueda@mrc-bsu.cam.ac.uk (O.M.R.), cc234@cam.ac.uk (C.C.)
https://doi.org/10.1016/j.xcrm.2025.102450

SUMMARY

The tumor microenvironment (TME) contributes to breast cancer heterogeneity and outcome but is rarely considered in clinical decision-making. We address this gap by systematically characterizing the TME's cellular composition to establish its independent clinical utility across intrinsic and genomic subtypes. We first compare 15 TME profiling methods in 693 samples and then apply the deconvolution algorithm InstaPrism to a meta-dataset of 14,837 expression profiles. We identify seven distinct TME patterns that associate with disease-free survival independently of intrinsic subtype. We also identify TME features that modulate chemotherapy response, relapse, and metastatic risk, with divergent patterns observed across estrogen receptor subtypes. Notably, long-term recurrence was regulated by vascular stromal cells and the innate immune response. Furthermore, the depletion of B cell lineage derivatives in metastatic lesions suggests an opportunity for therapeutic intervention. These results provide evidence for using TME characterization as a prognostic and predictive biomarker and identify potential targets for TME-based intervention.

INTRODUCTION

Breast cancer (BC) is a highly heterogeneous disease, driven by genomic changes that stratify the disease into subtypes with distinct pathogenesis, clinical outcomes, and treatment responses.[1–3] The tumor microenvironment (TME) is composed of non-cancer cells within the tumor, such as blood vessels, innate and adaptive immune cells, cancer-associated fibroblasts (CAFs), and normal epithelial cells. The BC TME plays a crucial role in disease progression and treatment response, shaped by dynamic interactions among its components, which can either promote or suppress tumor growth, depending on the disease context.[4–6] For instance, tumor-infiltrating lymphocytes are predictive of neoadjuvant treatment responses and improved overall survival in triple-negative BC and human epidermal growth factor receptor 2 (HER2)-positive BC but are associated with adverse survival in luminal estrogen receptor (ER)-positive and HER2-negative BC.[7–9] The characterization of the TME thus represents an opportunity to enhance risk stratification and optimize treatment strategies when combined with existing molecular stratification methods, which tend to be mostly based on the genomic aberrations of the malignant cells.

Most studies of the TME have been limited in scope, often focusing on just one or two specific cell types.[8,10–14] Among the few studies that have provided a more comprehensive characterization of the TME, most have excluded non-immune components such as vessels and CAFs due to technical constraints.[7,15] This is significant as vessel cell and CAF heterogeneity seem to have clinical and biological implications.[16–18] To better understand the diversity of the BC TME and inform the rational development of treatments and tools for patient management, it is necessary to enumerate TME features in a way that accounts for the full range of their specialized functions.[4] This requires considering the TME within large patient cohorts

© 2025 The Author(s). Published by Elsevier Inc.
This is an open access article under the CC BY license (http://creativecommons.org/licenses/by/4.0/).

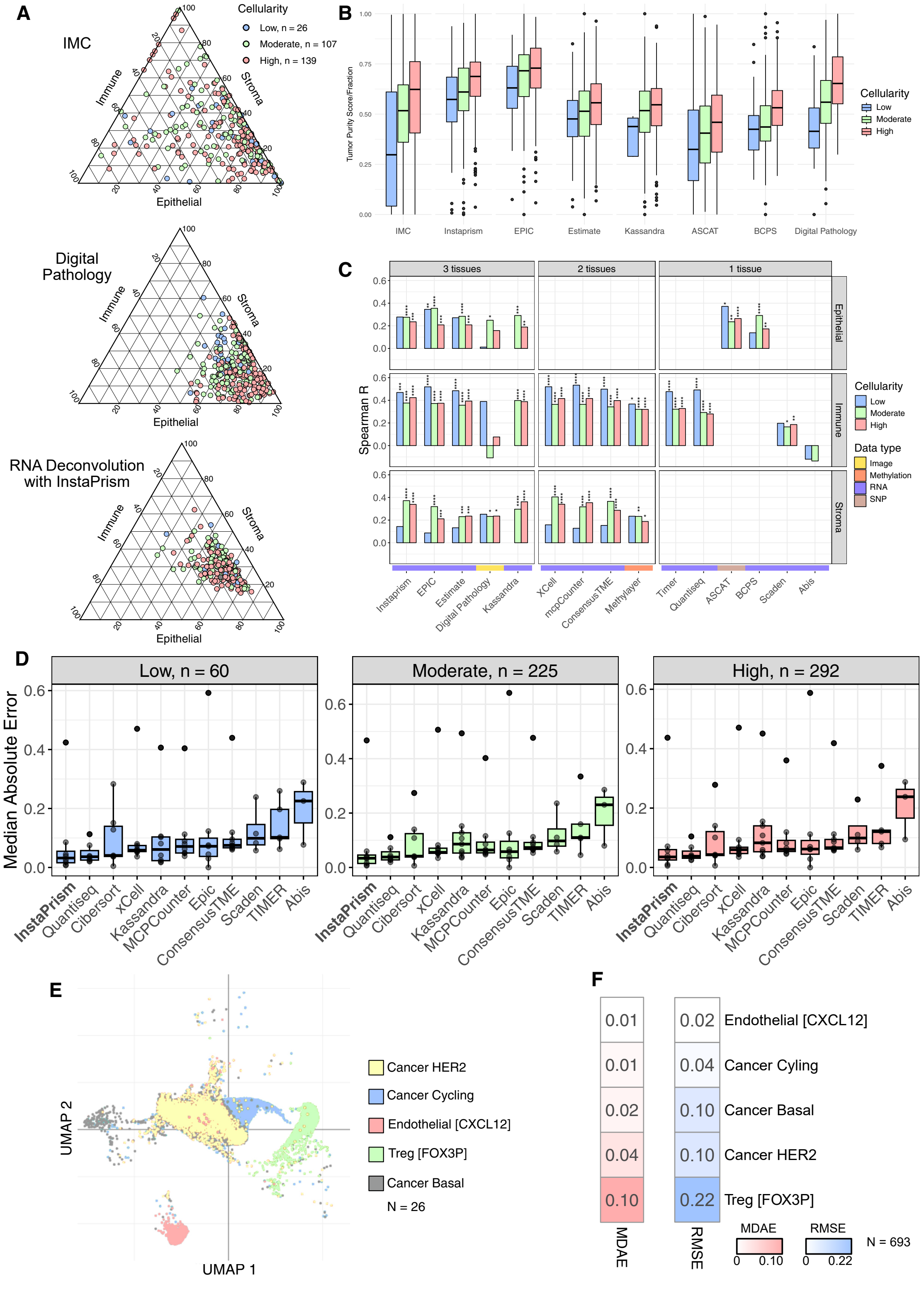

Figure 1. Comparing TME characterization methods

(A) Ternary plots of TME profiles using different data modalities, *n* = 272.

(B) Cellularity levels as determined by different methods across the different pathologist-graded tumor purity categories, scaled to a range of 0–1 for inter-method comparability.

(legend continued on next page)

CellPress
OPEN ACCESS

that reflect the molecular diversity of the disease. Recent studies have shown that the cellular composition of the TME can be grouped into distinct, recurring patterns, termed "ecotypes," which have been associated with clinical outcomes in cancer.[17,19]

Cell type deconvolution from bulk measurements and digital pathology are potential methods for characterizing the TME at scale. Cellular deconvolution is a process used to extract cell type information from bulk genomic data. Deconvolution identifies specific types of cells in a particular tissue based on an RNA signature expression, DNA methylation, and/or the genomic profile of several genes for each cell type. Digital pathology uses machine learning to classify cellular components based on image analysis of tissue sections. The algorithms are trained using labels from a subset of the images classified by an expert pathologist.[20]

The aims of our study were 2-fold. We first sought to select a robust TME characterization method by comparing several approaches in a cohort of 693 patients with BC analyzed with multiple profiling modalities. We then applied the most parsimonious algorithm to a meta-dataset of RNA expression profiles from 14,837 patients with BC and identified associations between the TME and clinical features. We observed interactions between TME features and molecular subtypes, as well as TME features associated with prognosis, relapse, treatment response, and metastases. These findings have the potential to inform the development of better biomarkers for patients with BC.

RESULTS

Selection of a TME characterization method

We used three methods to characterize the TME of BCs in a set of samples from the METABRIC cohort.[2,3]

(1) Imaging mass cytometry (IMC): this identifies cell types based on protein marker expression. Data were analyzed in 749 images corresponding to 693 tumors.
(2) Digital pathology: this identifies cell types using cell morphology data from hematoxylin and eosin-stained slides digitized as images, available for 564 tumors.
(3) Deconvolution: this estimates cell type composition using signatures derived from RNA expression, DNA single-nucleotide polymorphism/copy number, or DNA methylation data. Fourteen different deconvolution algorithms were applied to 1,980 tumors.

TME characterization using different data modalities may produce varying results for the same tumor, and it remains unclear which modality provides the most accurate assessment. The TME can be categorized into three main tissue types: immune, epithelial, and stromal. We first compared how each data modality assesses the proportions of these main cell types in tumors for which we had matched data from the three modalities. The proportions of the three TME cell types determined by each method differed (Figure 1A). The proportions determined through IMC were more variable across samples compared to InstaPrism (a deconvolution algorithm, selected from all 14 methods to illustrate features of RNA expression deconvolution-based methods), and digital pathology tended to have higher epithelial and lower stromal estimates (Figure 1A). Interestingly, despite the variability between methods, each approach appeared to estimate TME components in a way that aligned with expected values. For example, epithelial tissue values increased across different tumor cellularity bins for all methods assessing epithelial components, which mirrored the tumor cellularity classifications made by pathologists (Figure 1B).

To guide our method selection, we used IMC as a reference group, as it provided spatially resolved single-cell protein expression data. Although IMC was more variable than some other modalities and reflected only a single tissue region—thus potentially missing tumor-wide heterogeneity—we found that inter-patient variation in the TME was greater than intra-patient sampling variation (Figures S1–S3). Among the 59 tumors with multiple IMC images, the average within-sample Bray-Curtis dissimilarity was 0.29 (Figures S4A and S4B). In contrast, the average dissimilarity between different tumors was 0.38 (Figure S4C). These results suggest that while sampling variation within a tumor contributes to overall heterogeneity of the dataset, it is outweighed by differences between patients. We reasoned that this, in addition to having a relatively large sample size of 693 patients from which IMC data were collected, justified the use of IMC as a reference group for our benchmarking analysis.

Some algorithms were limited to deconvolving the TME at the tissue level (digital pathology, ASCAT, BCPS, MethyLayer, and ESTIMATE). At this granularity, we identified significant but weak-to-moderate correlations between IMC and the different TME analysis methods (Figure 1C). Generally, we found that RNA-based deconvolution methods had the highest correlation to IMC (Pearson r = 0.2–0.5) across both cell type and cellularity categories, factors which have been shown to affect deconvolution performance.[21] These moderate correlations likely arise because IMC captures localized regions of the tumor, while gene expression-based algorithms reflect the overall composition of the entire tumor. At the cell level, we used median absolute error (MDAE) to represent deconvolution accuracy. While many expression-based methods performed similarly, InstaPrism showed marginally lower MDAE across cellularity estimates (Figures 1D and S5). Root-mean-square error (RMSE) comparisons supported this conclusion (Figure S6). We also observed that fibroblasts were consistently the least accurately deconvoluted cell type across all TME characterization methods

(C) Bar plots of Spearman correlations between different TME characterization methods and IMC.
(D) Deconvolution performance of MDAE across pathologist-scored tumor cellularity.
(E) Uniform manifold approximation and projection of single-cell reference profiles of cell subtypes from deconvolution training dataset with matched IMC data.
(F) Deconvolution performance of InstaPrism across cell subtypes using MDAE (left, in red) and RMSE (right, in blue). *$p < 0.05$, **$p < 0.01$, ***$p < 0.001$, ****$p < 0.0001$.

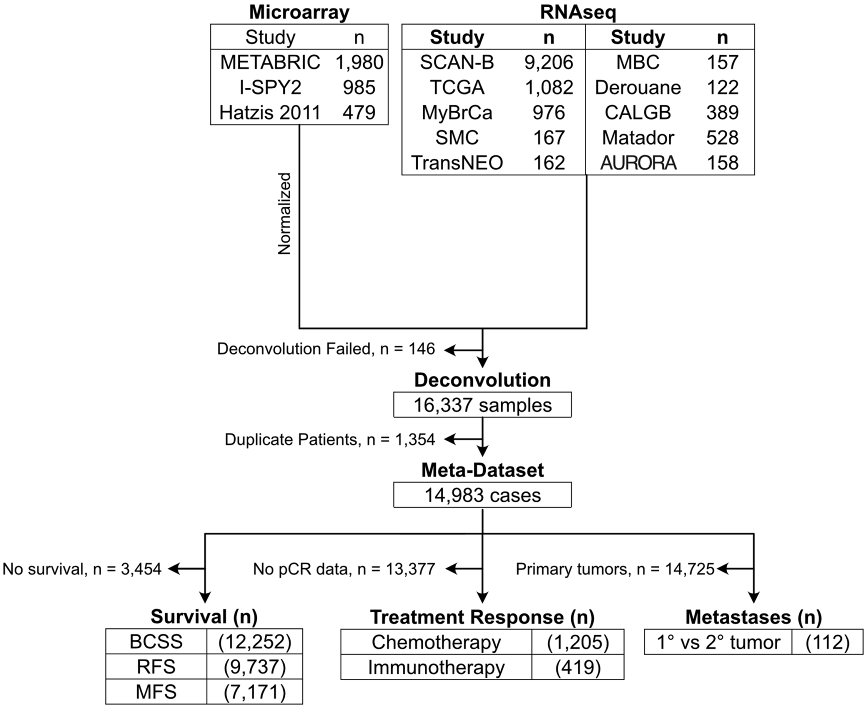

Figure 2. -dataset consort diagram

(Figure S5). As a result, the overall average performance of algorithms that did not deconvolve fibroblasts may be artificially inflated. We selected InstaPrism for downstream analyses based on its performance, the highest resolution among the selected methods, and computational efficiency.

We also evaluated the performance of InstaPrism at the cell-subgroup level. There were only 5 overlaps at the subgroup level between the IMC dataset and the single-cell training dataset used for InstaPrism, providing a limited sample size to test prediction quality. Within the single-cell RNA sequencing training data, the cancer epithelial groups in this list were transcriptionally similar (Figure 1E), Despite this, InstaPrism was able to predict cell subtypes with an MDAE less than or close to 0.1 and RMSE less than 0.22 (Figure 1F), mirroring its performance at the cell type level. Furthermore, InstaPrism can separate transcriptionally similar Cancer [Her2] and Cancer [Basal] and cycling cancer populations with MDAE 0.01–0.04 and RMSE 0.04–0.10 (Figure 1F). These results suggest that deconvolution at the cell subtype level is also relatively robust.

A meta-dataset of 14,837 BCs

We compiled a large, clinically annotated, transcriptome meta-dataset comprising 14,837 patients from 13 different studies (Figure 2A; Table S1). Using InstaPrism, we deconvoluted this dataset and investigated associations between the TME and various clinical features. Overall, the cohorts included 72.7% of patients with ER+ BC and 14.2% with HER2+ BC, and 82.5% of patients were between the ages of 41 and 80 (Table S2). Most tumors were grade 2 (47.3%) and stage 1 disease (50.2%) (Table S2).

In compiling this meta-dataset, we considered the potential impact of batch effects. InstaPrism transforms an expression matrix into compositional data by assessing the relative expression of cell type markers within each study. As batch effects are transformations of what should be similar expression patterns for the same disease, the deconvolution algorithms should inherently reduce their influence (Figure S7A). Indeed, based on principal component analysis of the ten RNA sequencing (RNA-seq) studies included in the meta-dataset, the confidence ellipses for eight studies overlapped with that defined by the remaining studies, suggesting evidence of batch effect mitigation in some RNA-seq studies (Figure S7B). The two studies that separated, AURORA and Derouane, may be explained by biological and technical differences (Figure S7B). Namely, the AURORA study comprises metastatic BC, and the Derouane study extracted RNA from formalin-fixed paraffin-embedded samples.

In contrast, microarray studies were clustered primarily by batch, likely due to differences in tumor sampling methods, array technologies, and probe selection (Figure S7C). For example, I-SPY2 collected samples using fine needle aspiration or core needle biopsies, whereas METABRIC relied on fresh tumor resections. Furthermore, although InstaPrism was originally developed for RNA-seq data, applying it to microarray data required additional transformation steps to meet the algorithm's input requirements. However, the strong concordance between InstaPrism's predictions and those from microarray-based methods suggests that this technical difference was unlikely to be the primary driver of batch separation (Figures S2–S4).

To preserve the biological differences in the compiled dataset, we avoided applying a batch-effect correction algorithm.[22] Instead, we stratified the analyses by study in downstream analyses, when possible, to statistically account for batch effects. When this was not possible, we limited the analyses to the study with the largest sample size available for the outcome of interest.

TME differences across molecular and genomic tumor subtypes

Tumor subtype and cellularity have been shown to influence cellular interactions within the TME, thereby shaping its composition. We compared TME composition across BC subtypes, stratified by ER and HER2 status and by tumor cellularity. Tumor cellularity was quantified based on the percentage of tumor cells estimated from InstaPrism, and samples were grouped into quartiles for comparison. Dendritic cells (DCs), B cells, and CAFs generally trended downward across cellularity quartiles, whereas

myeloid, endothelial, and perivascular-like (PVL) cells generally trended upward across cellularity quartiles (Figure 3A). T cells and normal epithelial cells had differing trends depending on tumor subtype. Interestingly, we observed increasing T cell proportions in ER+/HER2− tumors—defined largely by luminal A tumors—unlike other subtypes (Figure 3A). This seemed to be driven primarily by type 1 interferon T cells and T follicular helper cells (Figure 3B). We also noticed higher B cell proportions in ER−/HER2+ tumors with lower tumor cellularity, mostly driven by an abundance of memory B cells and plasmablasts, a type of rapid antibody-producing plasma cell (Figures 3A and 3D). We noticed similar TME trends within the expression-based Pam50 subtypes (Figure S8A), providing further evidence for the observation that tumor subtype and tumor cellularity correlate with differences in adaptive immune response.

We did a similar analysis across the integrative clusters and observed heterogeneity in the TME across the genomic subtypes that seemed to associate with tumor cellularity[2,3] (Figure S8B). CAFs and myeloid populations showed the greatest variability across integrative clusters, generally displaying an inverse relationship (Figure 3D). For example, IntClust 10, which primarily includes triple-negative BCs, had significantly lower CAF levels compared to IntClusts 3 and 5 but significantly higher myeloid infiltration compared to IntClusts 3, 7, and 8. Similarly, IntClust 3, which had the highest average CAF abundance, exhibited the lowest average myeloid population (Figure 3E). Endothelial cells increased with tumor cellularity across nearly all integrative clusters, consistent with patterns of angiogenesis, with the exception of IntClust 4ER−. T cell and B cell populations generally remained stable or decreased as cellularity increased. CAFs either peaked at intermediate levels of cellularity before declining or showed a steady downward trend. While we highlight high-level trends in cell populations for selected genomic subtypes of BC, the dataset also serves as a valuable resource for exploring other associations including at the cell subtype level.

Clustering BCs based on TME composition is an independent prognostic biomarker

We investigated whether distinct TME composition patterns based on 37 InstaPrism-deconvoluted cell phenotypes could stratify BCs into biologically meaningful groups. To minimize confounding from batch effects, this analysis was restricted to the 7,868 tumors from the SCANB cohort. Using *k*-means clustering and expectation maximization with Bayesian Information Criterion, we identified eight recurrent TME cell clusters and seven distinct TME composition clusters, which we term "TME types."

The eight cell clusters represent common patterns of immune, stromal, and vascular cell infiltration.

(1) Cell cluster 1: activated antigen presentation and T cell proliferation indicate ongoing immune activation, possibly early in response.
(2) Cell cluster 2: naive adaptive immunity and epithelial homeostasis suggesting resting or initiation-phase immune state with possible early-stage immune surveillance.
(3) Cell cluster 3: stromal remodeling and vascular niche indicative of stromal activation and vascular remodeling.
(4) Cell cluster 4: inflammatory monocyte response suggests innate immune response often seen in pre-immunosuppressive TME stages.
(5) Cell cluster 5: cytotoxic and innate effector activation—a highly cytotoxic and inflamed immune landscape associated with immune "hot" tumors.
(6) Cell cluster 6: immunosuppressive myeloid program reflecting a tumor-promoting milieu.
(7) Cell cluster 7: humoral immunoregulation potentially supporting B cell maturation while tempering immune activation.
(8) Cell cluster 8: proliferative vascular-stromal expansion possibly supporting metastasis, immune escape, or rapid tumor growth.

The TME types were enriched with the following InstaPrism cell types (Figure 4A; Table S3).

(1) TME type 1 (T1): stromal-vascular enriched with low adaptive immunity.
(2) TME type 2 (T2): immune-inflamed with active cytotoxic and regulatory cells.
(3) TME type 3 (T3): immunosuppressive with vascular remodeling and B cell support.
(4) TME type 4 (T4): dual-enriched immunosuppressive and cytotoxic program.
(5) TME type 5 (T5): stromal-immunosuppressive with reduced effector activity.
(6) TME type 6 (T6): stromal-proliferative with minimal immune infiltration. Similar to T5 but with lower tumor cellularity.
(7) TME type 7 (T7): naive-epithelial dominant with low stromal and myeloid content.

The TME types correlated with clinical characteristics (Figure 4B). T1 and T7 included tumors of lower stage and grade, T5 included tumors of intermediate stage and grade, and T4 included tumors of higher stage and grade. T2, T3, and T6 had tumors with mixed patterns of stage and grade. The TME types were also significantly enriched for PAM50 intrinsic subtypes. T1 was enriched with LumA and normal subtypes, T2 was enriched with Her2 and LumB, T3 was enriched with LumB, T4 was enriched with basal and Her2 subtypes, and T6 and T7 were enriched with LumA (Figure 4C).

The TME types independently predicted relapse-free survival (RFS) across the PAM50 intrinsic subtypes (Figure 4D). T4 had the worst prognosis in LumA and LumB tumors; T3 and T5 consistently had the worst prognosis in the basal subtype, and this was probably due to its low proportion of immune cells and high proportion of stromal and immunosuppressive macrophages (Figure 4D). T6 generally had the best prognosis. While T5 and T6 were similar, TME T6 tended to comprise lower-cellularity tumors (Figure 4A). The TME types showed greater separation of survival in basal and HER2 tumors, suggesting that TME features are more relevant in BC subtypes with poorer survival outcomes. To validate the TME types, we applied a random forest classifier to assign TME types within The Cancer Genome

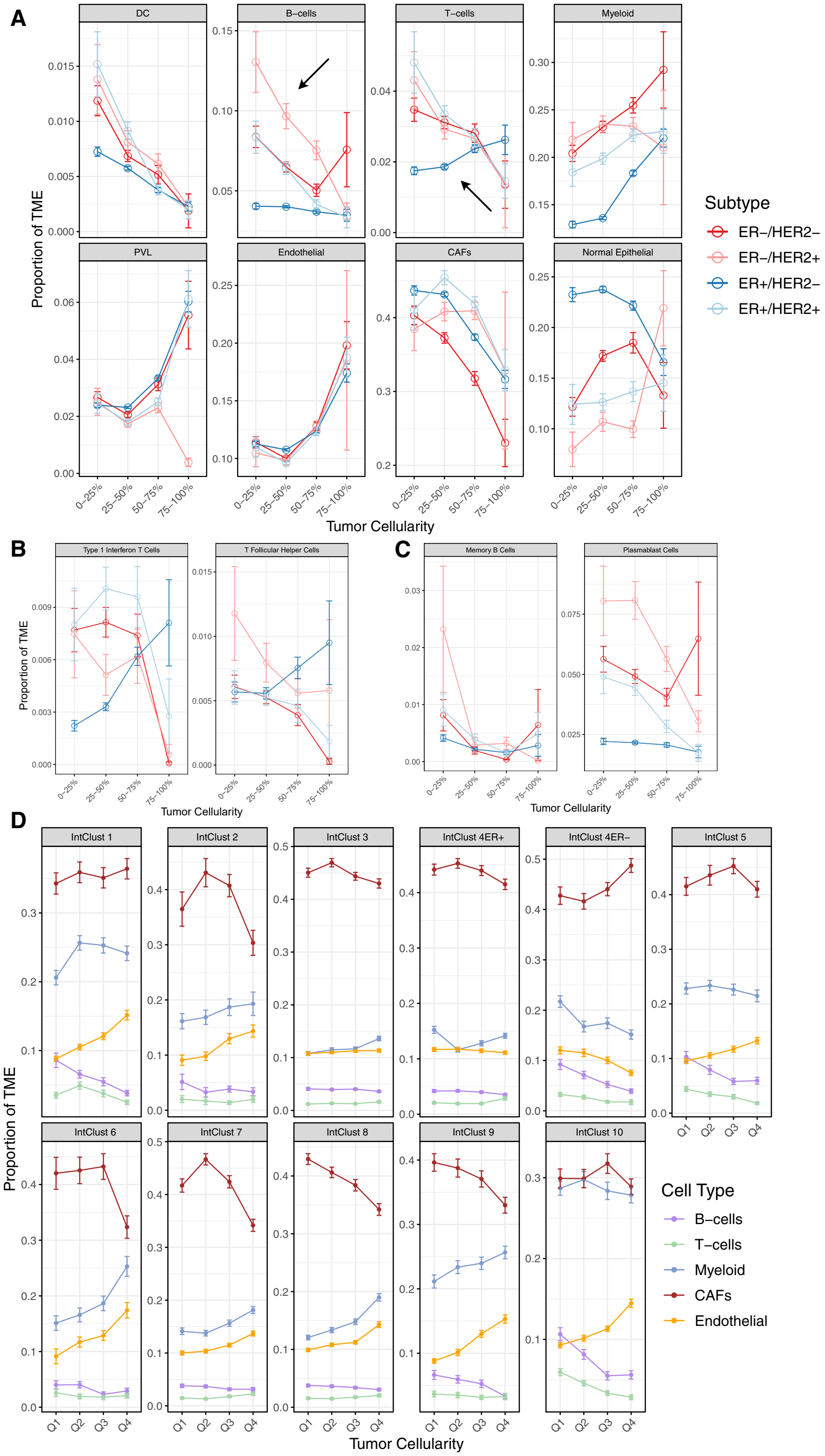

Figure 3. TME composition differences across BC subtypes and different quartiles of tumor cellularity

(A) Comparing cell types between ER/HER BC subtypes over cellularity quartiles reveals notable differences in T cell, endothelial, and B cell populations.

(B) Comparing T cell subtypes between ER/HER BC subtypes over cellularity quartiles.

(C) Comparing B cell lineage subtypes between ER/HER BC subtypes over cellularity quartiles.

(D) Cell type proportions across IntClusts. All error bars represent SEM. All cell types and subtypes derived from InstaPrism training set.

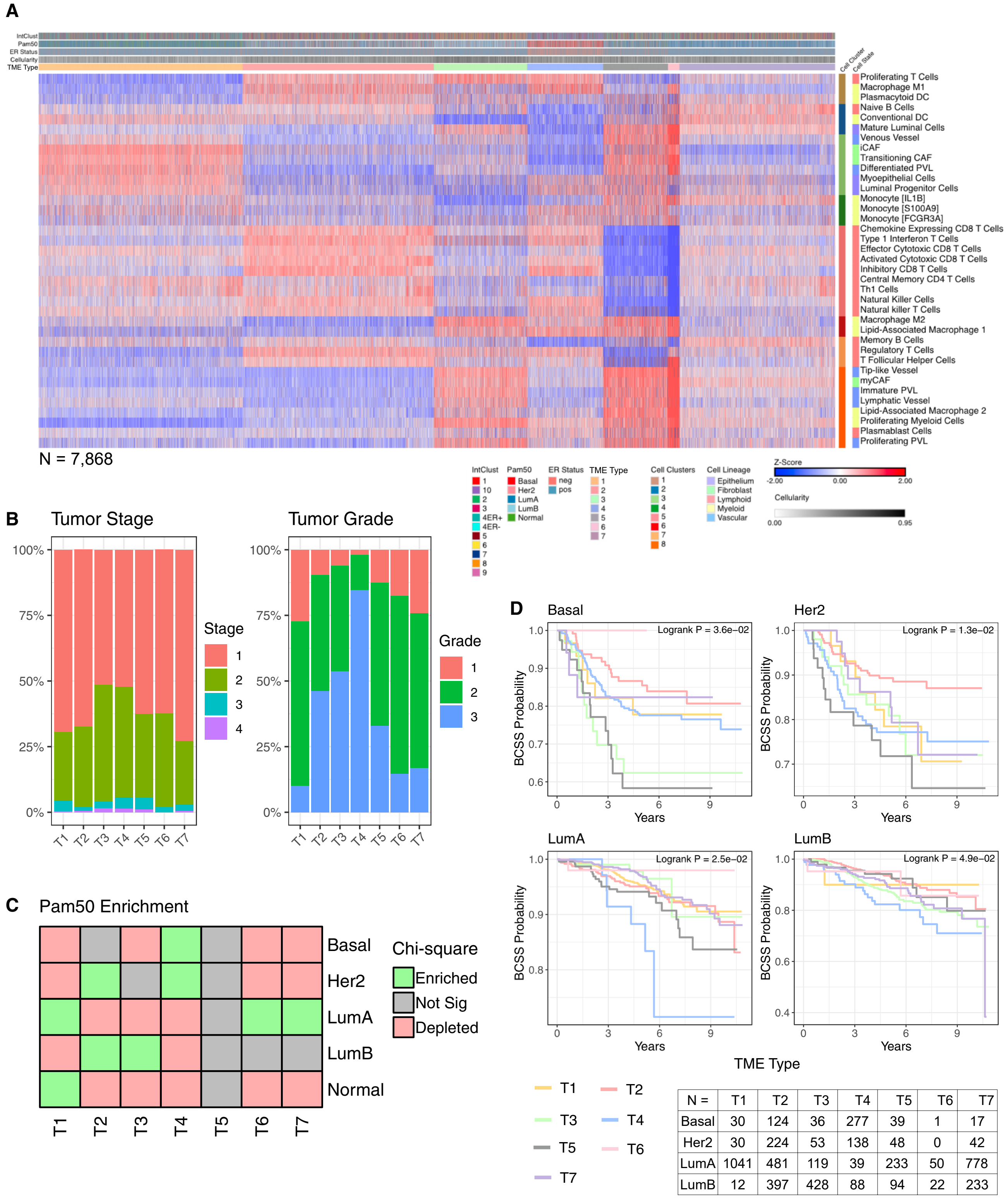

Figure 4. TME types complement genomic stratification of BC

(A) Heatmap of InstaPrism cell type proportions using *k*-means clustering. *n* = 7,868 tumors (SCANB).

(B and C) Stacked bar charts of tumor stage and grade prevalence across the TME types.

(D–F) Kaplan-Meier curves of RFS for TME types across selected PAM50 subtypes with at-risk tables, log rank test comparing the survival between clusters.

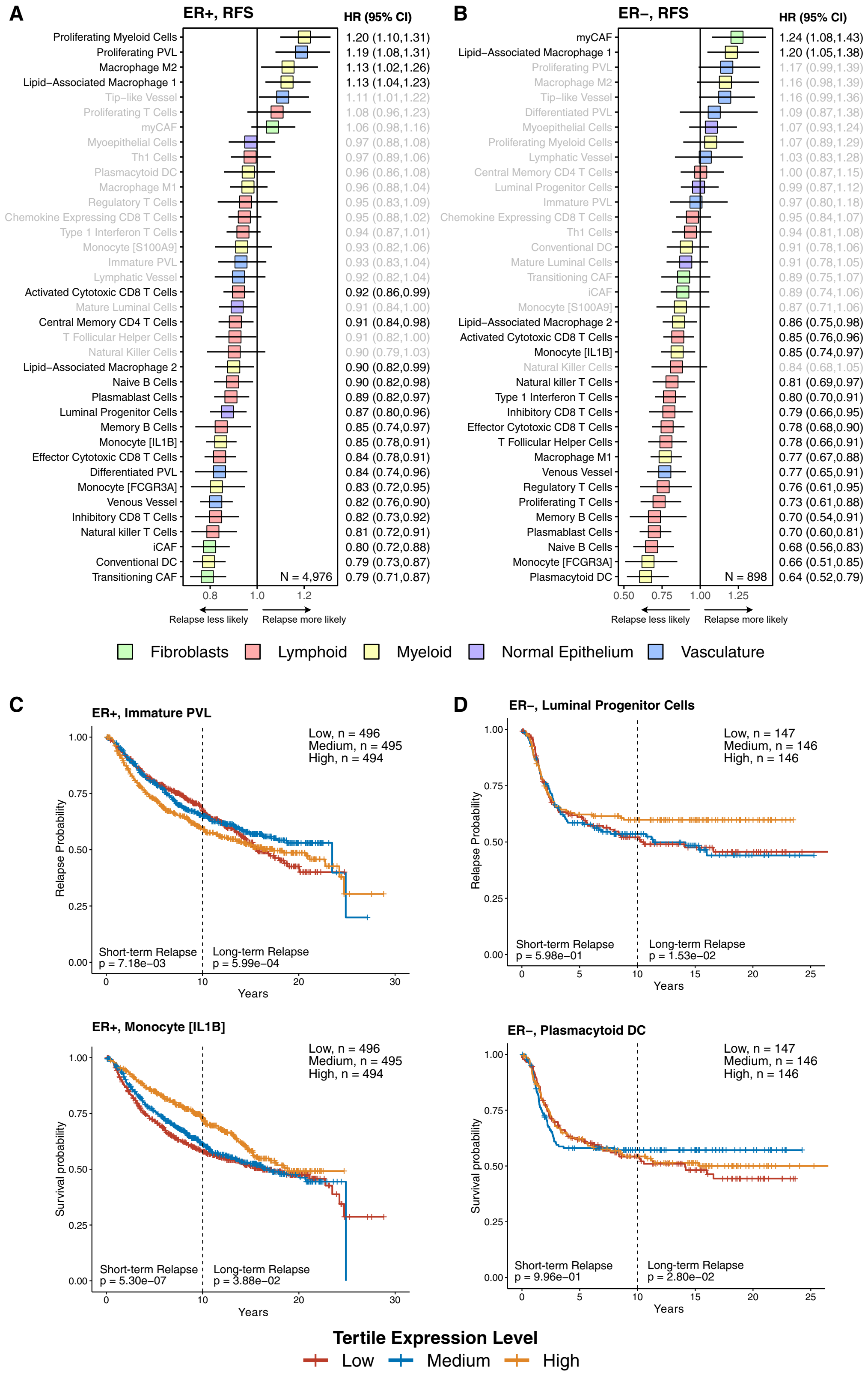

(legend on next page)

Atlas (TCGA) cohort. Among the luminal tumors, which had sufficient sample sizes for meaningful comparison, we observed similar survival patterns to those seen in the SCANB cohort (Figure S9). For instance, T4 was again associated with the worst survival in luminal B tumors. These results indicate that distinct TME types could serve as independent prognostic markers, complementing the clinical subtypes currently used.

Associations between InstaPrism cell types and BC relapse

To identify TME features associated with risk of relapse, we performed multivariable Cox proportional hazards modeling of RFS in ER− and ER+ tumors, adjusting for tumor stage, grade, nodal involvement, and study-specific variability. Our results demonstrated that a heterogeneous mixture of cellular lineages was associated with relapse events in ER+ BC (Figure 5A; n = 4,976 from METABRIC, SCANB, and TCGA). Cells involved in the adaptive immune response such as DCs were associated with reduced relapse. In addition, inflammatory CAFs were also associated with less relapse, presumably by recruiting immune cells to the lesion. On the other hand, immunosuppressive M2 macrophages and lipid-associated macrophages 1 (LAM1) were associated with relapse (Figure 5A). Interestingly, in some cases, the developmental state of the cell was relevant; for instance, proliferating PVL cells—presumably less differentiated—were associated with relapse, whereas more differentiated PVL cells were associated with a lower likelihood of relapse (Figure 5A).

Compared to ER+ BC, the effectors of relapse in ER− BC were more homogenous as myeloid and T cells made up 89% of the features that had a significant association with RFS events (Figure 5B; n = 898 from METABRIC, SCANB, and TCGA). Myofibroblastic cancer-associated fibroblasts (myCAFs) and lipid-associated macrophages (LAM1s) were associated with relapse, while plasmacytoid and monocyte (FCGR3A) populations were inversely associated with relapse (Figure 5B). Interestingly, we also noted that regulatory T cells, which are typically immunosuppressive, were associated with less relapse in ER− tumors, consistent with previous large meta-analyses (Figure 5B).[23] Notably, LAM1 and LAM2 were linked to relapse and reduced relapse, respectively, in both ER+ and ER− tumors. As a recently discovered BC cell type, the functions of LAM1 and LAM2 remain unclear. However, our findings suggest that they may have opposing roles in modulating relapse, with LAM1 potentially acting as an immunosuppressive factor.

To validate these findings, we performed multivariable Cox proportional hazards modeling using BC-specific survival (BCSS). Consistent with our analysis of RFS, many of the same TME features were associated with BCSS outcomes (Figure S10A). Of note, when further stratifying by PAM50 intrinsic subtype, we found that the prognostic influence of the TME was strongest in the poor-prognosis basal and Her2 subtypes, whereas luminal A, luminal B, and normal-like tumors showed little association between TME composition and survival (Figure S10B). These results suggest that TME-targeted therapies and monitoring may have the greatest clinical utility in aggressive BC subtypes.

Given the propensity for late relapse in BC—particularly in ER+ disease, where recurrence often occurs after a decade of clinical dormancy—we sought to evaluate whether specific TME features were associated with time-dependent risk of relapse. Conventional Cox models assume constant hazard over time and may fail to capture dynamic risk factors that govern long-term dormancy and relapse. To address this, we analyzed ER+ and ER− tumors separately within the METABRIC cohort—the only dataset in our meta-analysis with sufficient long-term follow-up to enable this analysis. For each immune and stromal cell type, we stratified tumors into tertiles of cell type enrichment and generated Kaplan-Meier survival curves. To assess whether the impact of TME features differed between early and late relapse, we performed time-stratified log rank tests. Short-term relapse risk ($\leq$10 years) was assessed using survival data censored at 10 years, while long-term relapse risk (>10 years) was evaluated using left-truncated survival analysis restricted to patients who remained relapse-free beyond 10 years.

In ER+ tumors, low enrichment of immature PVL cells correlated with elevated short-term relapse risk ($p = 7.18 \times 10^{-3}$) but was paradoxically protective against long-term recurrence ($p = 5.99 \times 10^{-4}$), marking PVL as the sole TME feature with a temporal risk-switching effect (Figure 5C). Conversely, high monocyte (IL1B) enrichment initially conferred protection against long-term relapse ($p = 5.30 \times 10^{-7}$), though this association attenuated over time ($p = 3.88 \times 10^{-2}$), suggesting time-dependent roles for IL1B$^+$ monocytes in modulating immunosurveillance. These findings implicate immature PVL dynamics and transient monocyte (IL1B) activity as key mediators of ER+ BC recurrence.

In ER− tumors, long-term relapse mechanisms diverged. High luminal progenitor cell enrichment correlated with reduced long-term relapse risk ($p = 1.53 \times 10^{-2}$), while extreme tertiles (low or high) of plasmacytoid DCs were associated with elevated recurrence ($p = 2.80 \times 10^{-2}$) (Figure 5D). This bimodal effect may be consistent with the dual roles of plasmacytoid DCs, wherein low levels limit immune recruitment, while high levels promote chronic inflammation that can promote tumor growth.[24]

Collectively, these data demonstrate that the cellular composition of the TME differentially associates with the temporal kinetics of relapse in ER+ and ER− BC. Long-term ER+ recurrence is associated with temporally dynamic interactions with PVL cells and IL1B$^+$ monocytes, whereas ER− relapse is associated

Figure 5. The effects of TME features on relapse

(A) Adjusted Cox model in ER+ patients for RFS, n = 4,976 (METABRIC, SCANB, and TCGA).

(B) Adjusted Cox model in ER− patients for RFS, n = 898 (METABRIC, SCANB, and TCGA).

(C) Kaplan-Meier curves of immature PVL and monocyte (IL1B) cell states in ER+ tumors, with log rank p values for both short- and long-term relapse.

(D) Kaplan-Meier curves of luminal progenitor cells and plasmacytoid DCs in ER− tumors, with log rank p values for both short- and long-term relapse. RFS, relapse-free survival; HR, hazard ratio; CI, confidence interval. All cell types and subtypes derived from InstaPrism training set.

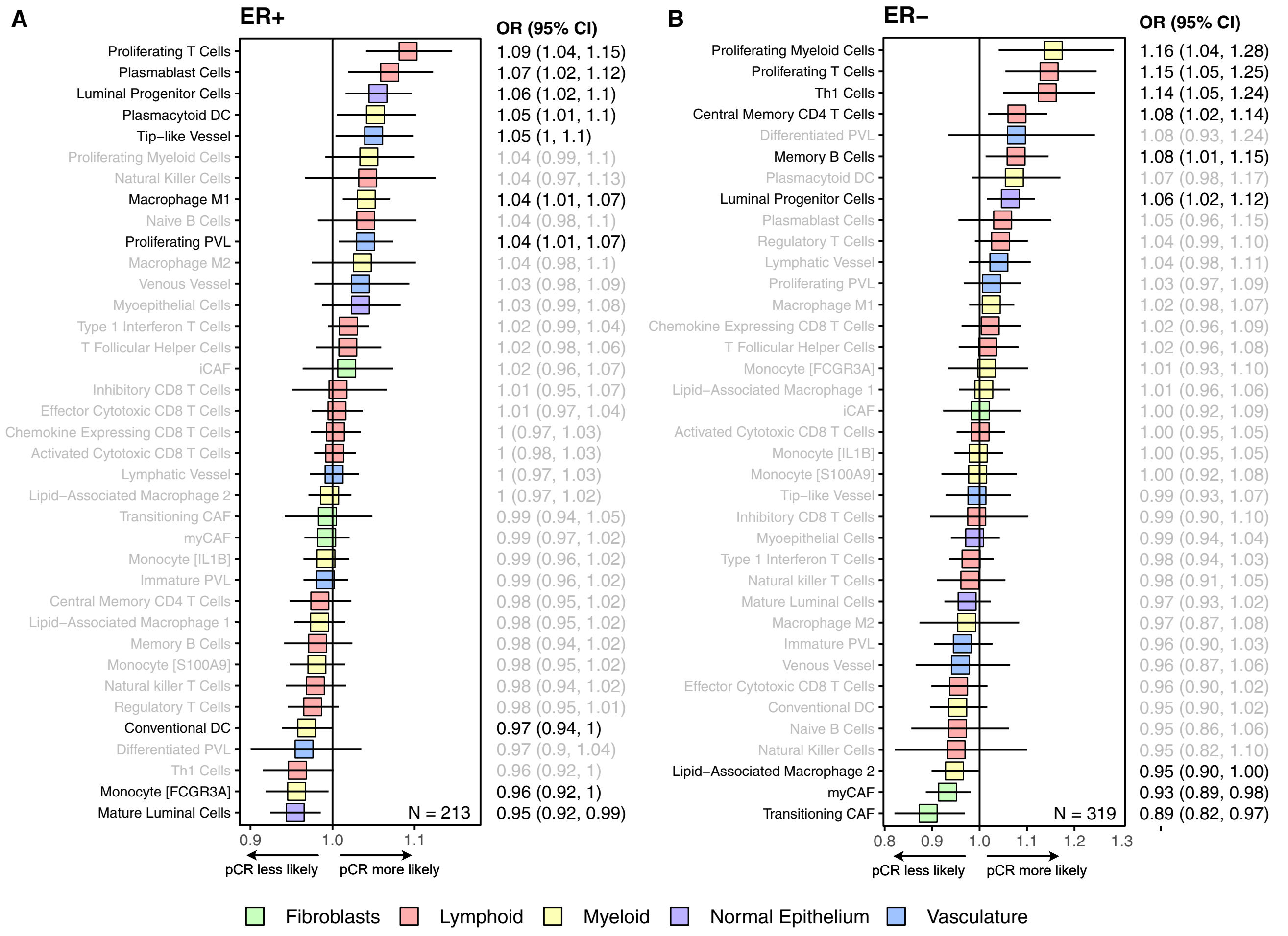

Figure 6. TME variables associated with chemotherapy response

(A) Logistic regressions of pCR to chemotherapy in ER+ patients, n = 213 (Hatzis 2011, I-SPY2, and TransNEO).

(B) Logistic regressions of pCR to chemotherapy in ER− patients, n = 319 (Hatzis 2011, I-SPY2, and TransNEO).

with luminal progenitor cell proportion and a bimodal effect of plasmacytoid DCs. These associations suggest that the TME landscape, in a BC subtype-specific manner, modulates the temporal shape of BC recurrence.

Associations between TME composition chemotherapy and immunotherapy response

We analyzed the association between TME cell type proportions in pre-treatment biopsies and response to neoadjuvant chemotherapy assessed by pathology at the time of surgery. We found that proliferating T cells and luminal progenitor cells are strongly associated with pathological complete response (pCR) in both ER+ and ER− BC (Figures 6A and 6B; ER+ n = 213 from Hatzis 2011, I-SPY2, and TransNEO; ER− n = 319 from Hatzis 2011, I-SPY2, and TransNEO). In contrast, transitioning CAFs and myCAFs were associated with less pCR in ER− BC (Figure 6B). We noted that in ER+ BC, plasmablasts, plasmacytoid DCs, and M1 macrophages (cells involved in rapid immune activation) were associated with pCR, whereas conventional DCs (involved in long-term adaptive immunity) associated with less pCR (Figure 6A). In contrast, T helper 1 cells, central memory CD4 T cells, and memory B cells (involved in adaptive immunity) were associated with pCR in ER− BC (Figure 6B).

TME, risk of metastases, and metastatic cellular composition

To identify TME features associated with risk of metastases, we performed multivariable Cox proportional hazards modeling of metastatic-free survival in ER− and ER+ tumors, adjusting for tumor stage, grade, nodal involvement, and study-specific variability. Proliferating myeloid cells and myCAFs were associated with metastases in ER+ and ER− BC, respectively (Figures 7A and 7B; ER+ n = 4,167 from SCANB; ER− n = 755). Effector cells or cells that contribute to effector functions of the adaptive immune system were strongly associated with less metastasis in both ER+ and ER− BC, such as plasmablasts, conventional DCs, natural killer T cells, naive B cells, and effector CD8 T cells. Stromal populations, including transitioning CAFs, venous vessels, luminal progenitors, and differentiated PVL

CellPress
OPEN ACCESS

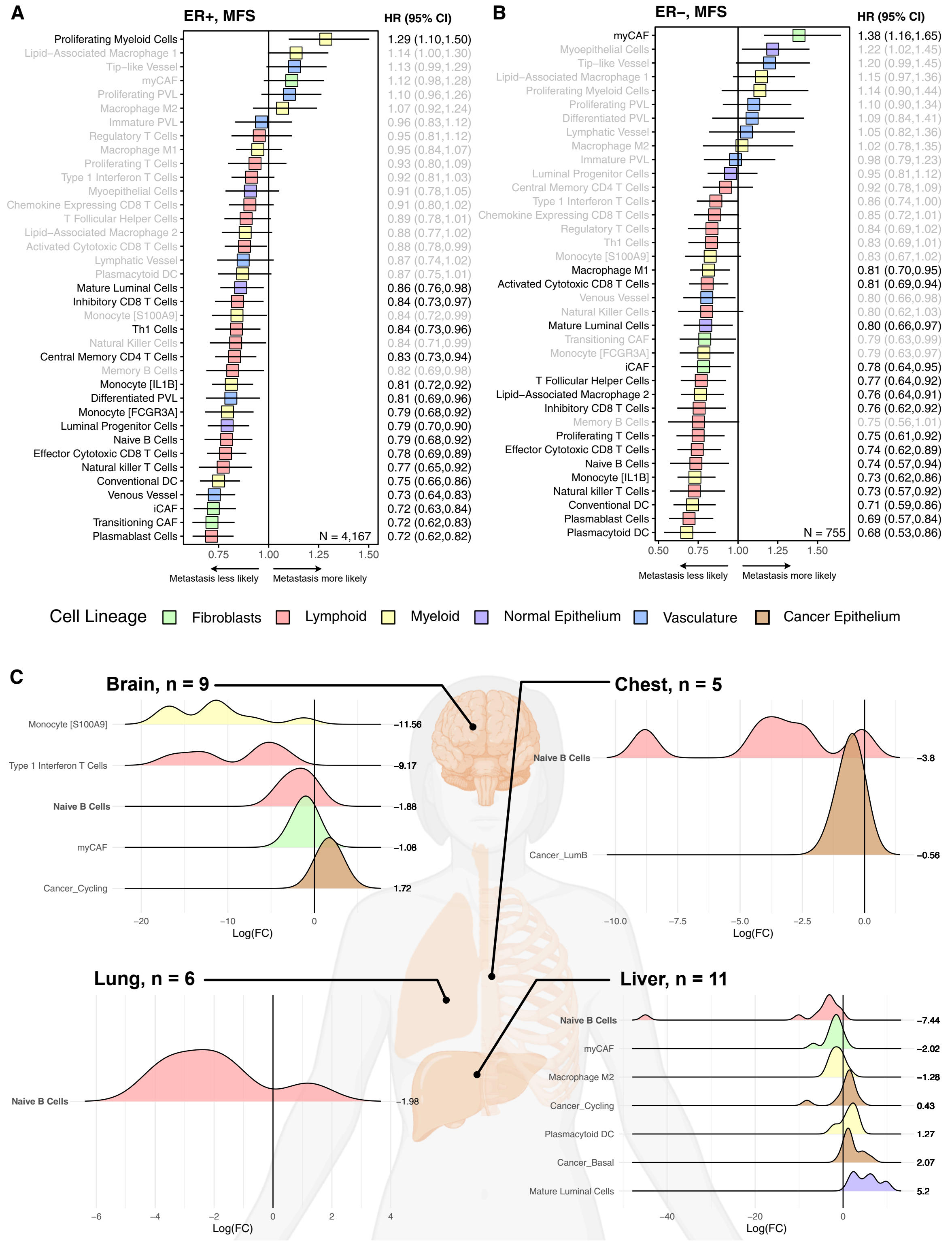

Figure 7. Metastatic risk and TME changes in metastatic samples

(A) Adjusted Cox model in ER+ patients for MFS, n = 4,167 (SCANB and Hatzis 2011).

(B) Adjusted Cox model in ER− patients for MFS, n = 755 (SCANB and Hatzis 2011).

(C) Significant differences in TME composition in paired metastatic and primary tumor samples (t test with Benjamini Hochberg correction, adj$P < 0.05$, AURORA). MFS, metastases-free survival; aHR, adjusted hazard ratio. ***$p < 0.001$, **$p < 0.01$, *$p < 0.05$, $p < 0.10$. All cell types and subtypes derived from InstaPrism training set.

cells, were associated with reduced metastasis in ER+ BC specifically. In contrast, inflammatory antigen-presenting cells such as plasmacytoid dendritic cells, M1 macrophages, and LAM2 populations were associated with less relapse in ER− BC.

We compared TME profiles between paired primary BCs and their distant metastatic lesions using the AURORA dataset.[25] While the differences we observed in the TME varied depending on the metastasis location, all sites showed significantly decreased levels of naive B cells (Figure 6C). Brain and liver metastases also had significantly fewer myCAF cells and significantly more proliferating cancer cells (Figure 6C), suggesting more aggressive tumors with higher cellularity.

DISCUSSION

We compared 15 TME characterization methods in 693 BC samples for which we had IMC, digital pathology, and deconvolution data. From this, we selected InstaPrism for its accuracy and granularity of output to deconvolute a cohort of nearly 14,837 BC tumors. Our findings reveal distinct TME composition patterns that independently predict survival, particularly in high-risk BC subtypes as well as complex clinical associations between TME cellular composition and relapse, treatment response, and metastasis. Notably, the B cell lineage emerged as associated with long-term relapse, chemotherapy and immunotherapy response, and metastases.

By clustering TME cell composition, we identified 7 TME types that were differentially enriched with overlapping PAM50 subtypes, suggesting that the TME organizes semi-independently of underlying tumor biology. The TME types separated prognostically within the PAM50 intrinsic subtypes, suggesting that TME features affect clinical outcomes in a highly context-dependent manner. This may be useful for further stratifying prognosis on top of the PAM50 schema. For instance, among the basal subtype, T3 was associated with poor BCSS, while T6 was associated with significantly better outcomes. Thus, characterizing the TME may complement existing BC clinical classifications.

While a direct one-to-one mapping to previously described BC ecotypes reported by Wu et al. is challenging due to differences in cohorts (SCANB vs. METABRIC), data modality (RNA-seq vs. microarray), and the deconvolution methods used (InstaPrism vs. CIBERSORTx), we observed notable similarities in the major biological themes.[17] For instance, our “immune-hot” T2 parallels their immune-rich ecotype 4, which is similarly defined by a high abundance of T cells. In contrast, our stromal-dominant T1, characterized by limited immune engagement, reflects their ecotype 2, which is defined by a cluster of mesenchymal cell types including CAFs and endothelial cells. A key distinction is that our TME types were defined after normalizing for cancer cell populations to isolate the TME’s independent prognostic value. In contrast, the Wu et al. ecotypes were clustered using both TME and cancer cell fractions, thus linking prognostic signals directly to the intrinsic cancer subtype. For example, their poorest-prognosis ecotypes, E3 and E7, are defined by an enrichment of aggressive basal-like and HER2-enriched cancer cells, respectively. Similarly, their most favorable-prognosis group, ecotype 2, is composed largely of less aggressive LumA and normal-like tumors. In contrast, our TME types were defined after normalizing for cancer cell populations. This approach was chosen specifically to isolate the prognostic power of the TME’s composition, independent of the intrinsic tumor subtype. This may explain why our stromal-dominant phenotypes such as T6 were associated with an immune-excluded state that suggests a poor prognosis, as they reflect the influence of the TME itself, rather than being confounded by the generally favorable prognosis of the luminal tumors they are enriched with.

Finally, our analysis highlights a recurring—and potentially underappreciated—role for B cell-mediated immunosurveillance in BC progression and metastasis. Across both ER+ and ER− tumors, higher levels of plasmablasts, naive B cells, and memory B cells were consistently associated with a reduced risk of relapse. These B cell lineages also correlated with treatment response as plasmablasts were enriched in chemotherapy responders in ER+ tumors, while memory B cells were linked to response in ER− tumors. Notably, naive B cells were uniformly depleted across all metastatic lesions, supporting the hypothesis that metastatic seeding cells may evolve to evade B cell-driven immune pressure. We also found that T helper 1 and T follicular helper cells—key regulators of B cell activation—were associated with reduced metastasis in both ER+ and ER− tumors. Their co-occurrence with protective B cell subsets suggests that effective immunosurveillance against metastasis may require coordinated B and T cell interactions. If metastatic progression involves evasion of B cell immunity, subsequent lesions may also lack both B cells and their supporting T cell partners within the TME. These observations point to the loss of B cell immunosurveillance as a potential enabling step in metastasis and raise the possibility that therapies aimed at restoring B cell function could help prevent or treat metastatic disease. However, given the limited sample size of the metastatic cohort, further studies will be necessary to validate these findings. We also acknowledge that using a reference derived from primary breast tumors to deconvolve metastatic samples has limitations, as metastatic sites may harbor stromal or immune cells with distinct transcriptional profiles. While this may affect the absolute accuracy for any single metastatic site, the consistent application of the method allows for the relative comparisons that were the focus of this analysis.

In summary, our study represents one of the largest efforts to link TME composition to clinical outcomes. We uncovered complex and distinct associations between specific TME cell populations and clinical endpoints, shaped by ER status and molecular subtype. These findings have potential utility for improving tumor prognostication, predicting relapse and treatment response, and guiding the development of tailored therapeutic strategies. As deconvolution algorithms continue to improve, their application to bulk tumor profiling offers a powerful and scalable approach to uncover the clinical significance of TME crosstalk. In the future, TME composition may serve as an independent biomarker for prognosis and therapy selection, complementing existing clinical and genomic markers, to better guide patient management and the development of more precise strategies to prevent relapse and metastasis.

CellPress
OPEN ACCESS

Limitations of the study

To identify a parsimonious algorithm suitable for our large meta-cohort, we used IMC as an orthogonal reference to benchmark various TME characterization methods. While IMC provides high-dimensional spatial data, it is limited by its sampling of single regions of interest, which may not fully capture intra-tumoral heterogeneity. This likely contributes to the modest correlations observed with bulk transcriptomic methods. However, because all methods were compared against the same IMC reference, relative performance assessments should remain valid. We also acknowledge that certain cell types—particularly CAFs—are inherently difficult to deconvolve due to their transcriptional heterogeneity. Nonetheless, our CAF- and immune-related findings were consistent across datasets and aligned with prior literature, supporting their biological plausibility.[26] We also addressed potential batch effects by including batch as a stratification variable in regression models when appropriate or by restricting analyses to the largest batch when necessary. Finally, we recognize the challenge of interpreting compositional data, where changes in one cell type's abundance may impact others. To mitigate this, we binned variables into quartiles and applied centered log-ratio transformations to project the data from the Aitchison simplex into Euclidean space. Despite these steps, our findings should be interpreted with caution and warrant downstream validation.

RESOURCE AVAILABILITY

Lead contact

Further information and requests for resources should be directed to and will be fulfilled by the lead contact, Kevin J. Tu (kevin.tu@som.umaryland.edu).

Materials availability

This study did not generate new unique reagents.

Data and code availability

- The processed datasets used for this manuscript are available at https://doi.org/10.17605/OSF.IO/B8VYT. Deconvolution methods and raw public datasets are available in the key resources table and further described in Tables S4 and S1.
- The code from this manuscript is available at https://doi.org/10.17605/OSF.IO/B8VYT.
- Any additional information required to reanalyze the data reported in this work paper is available from the lead contact upon request.

ACKNOWLEDGMENTS

The authors would like to thank Dr. Raza Ali and Dr. Tim Coorens for reviewing this work. We also extend our deepest gratitude to the patients whose data made this study possible. On a more personal note, we thank Dr. An Truong for paying for takeout during late nights in the lab and Dr. Noe Rodriguez for lending the author the bike that made it possible to get to work each day. Very special thanks to Angela Sun as well as the "Five Lads" who watched this work come together while traveling through two dozen different countries. This work was funded by UKRI grant MC_UU_00002/16. K.J.T. was funded by a Churchill Scholarship from the Winston Churchill Foundation of the United States, an AOA Carolyn L. Kuckein Research Fellowship, and the EACR Travel Grant. The funders had no role in study design, data collection and analysis, decision to publish, or preparation of the manuscript. For the purpose of open access, the author has applied a Creative Commons Attribution (CC BY) license to any Author Accepted Manuscript version arising.

AUTHOR CONTRIBUTIONS

K.J.T.: data curation, formal analysis, funding acquisition, investigation, methodology, software, validation, visualization, and writing – original draft. D.G.-R., K.E., and R.M.G.: software, visualization, and writing – review and editing. J.W. and S.-H.T.: data curation and writing – review and editing. L.N. and S.-J.S.: writing – review and editing. F.M.: supervision and writing – review and editing. O.M.R. and C.C.: conceptualization, funding acquisition, project administration, supervision, and writing – review and editing.

DECLARATION OF INTERESTS

The authors declare no competing interests.

STAR★METHODS

Detailed methods are provided in the online version of this paper and include the following:

- KEY RESOURCES TABLE
- EXPERIMENTAL MODEL AND STUDY PARTICIPANT DETAILS
- METHOD DETAILS
  - TME characterization
  - Benchmarking of characterization methods
  - Collation of *meta*-dataset
  - Identification of microenvironment signatures
  - Assessing disease-specific, relapse-free, and metastasis-free survival
  - Treatment response
  - Metastatic composition
- QUANTIFICATION AND STATISTICAL ANALYSIS

SUPPLEMENTAL INFORMATION

Supplemental information can be found online at https://doi.org/10.1016/j.xcrm.2025.102450.

Received: April 20, 2025
Revised: August 12, 2025
Accepted: October 14, 2025
Published: November 10, 2025

REFERENCES

1. Perou, C.M., Sørlie, T., Eisen, M.B., Van De Rijn, M., Jeffrey, S.S., Rees, C.A., Pollack, J.R., Ross, D.T., Johnsen, H., Akslen, L.A., et al. (2000). Molecular portraits of human breast tumours. Nature *406*, 747–752.
2. Curtis, C., Shah, S.P., Chin, S.F., Turashvili, G., Rueda, O.M., Dunning, M.J., Speed, D., Lynch, A.G., Samarajiwa, S., Yuan, Y., et al. (2012). The genomic and transcriptomic architecture of 2,000 breast tumours reveals novel subgroups. Nature *486*, 346–352.
3. Rueda, O.M., Sammut, S.J., Seoane, J.A., Chin, S.F., Caswell-Jin, J.L., Callari, M., Batra, R., Pereira, B., Bruna, A., Ali, H.R., et al. (2019). Dynamics of breast-cancer relapse reveal late-recurring ER-positive genomic subgroups. Nature *567*, 399–404.
4. Harris, M.A., Savas, P., Virassamy, B., O'Malley, M.M.R., Kay, J., Mueller, S.N., Mackay, L.K., Salgado, R., and Loi, S. (2024). Towards targeting the breast cancer immune microenvironment. Nat. Rev. Cancer *24*, 554–577.
5. Mayer, S., Milo, T., Isaacson, A., Halperin, C., Miyara, S., Stein, Y., Lior, C., Pevsner-Fischer, M., Tzahor, E., Mayo, A., et al. (2023). The tumor microenvironment shows a hierarchy of cell-cell interactions dominated by fibroblasts. Nat. Commun. *14*, 5810.
6. Gupta, P.B., Fillmore, C.M., Jiang, G., Shapira, S.D., Tao, K., Kuperwasser, C., and Lander, E.S. (2011). Stochastic State Transitions Give Rise to Phenotypic Equilibrium in Populations of Cancer Cells. Cell *146*, 633–644.

7. Bense, R.D., Sotiriou, C., Piccart-Gebhart, M.J., Haanen, J.B.A.G., Van Vugt, M.A.T.M., De Vries, E.G.E., Schröder, C.P., and Fehrmann, R.S.N. (2017). Relevance of Tumor-Infiltrating Immune Cell Composition and Functionality for Disease Outcome in Breast Cancer. J. Natl. Cancer Inst. *109*, djw192.
8. Denkert, C., Von Minckwitz, G., Darb-Esfahani, S., Lederer, B., Heppner, B.I., Weber, K.E., Budczies, J., Huober, J., Klauschen, F., Furlanetto, J., et al. (2018). Tumour-infiltrating lymphocytes and prognosis in different subtypes of breast cancer: a pooled analysis of 3771 patients treated with neoadjuvant therapy. Lancet Oncol. *19*, 40–50.
9. Sammut, S.J., Crispin-Ortuzar, M., Chin, S.F., Provenzano, E., Bardwell, H.A., Ma, W., Cope, W., Dariush, A., Dawson, S.J., Abraham, J.E., et al. (2022). Multi-omic machine learning predictor of breast cancer therapy response. Nature *601*, 623–629.
10. Ali, H.R., Provenzano, E., Dawson, S.J., Blows, F.M., Liu, B., Shah, M., Earl, H.M., Poole, C.J., Hiller, L., Dunn, J.A., et al. (2014). Association between CD8+ T-cell infiltration and breast cancer survival in 12 439 patients. Ann. Oncol. *25*, 1536–1543.
11. Adams, S., Gray, R.J., Demaria, S., Goldstein, L., Perez, E.A., Shulman, L.N., Martino, S., Wang, M., Jones, V.E., Saphner, T.J., et al. (2014). Prognostic Value of Tumor-Infiltrating Lymphocytes in Triple-Negative Breast Cancers From Two Phase III Randomized Adjuvant Breast Cancer Trials: ECOG 2197 and ECOG 1199. J. Clin. Oncol. *32*, 2959–2966.
12. Ali, H.R., Dariush, A., Provenzano, E., Bardwell, H., Abraham, J.E., Iddawela, M., Vallier, A.L., Hiller, L., Dunn, J.A., Bowden, S.J., et al. (2016). Computational pathology of pre-treatment biopsies identifies lymphocyte density as a predictor of response to neoadjuvant chemotherapy in breast cancer. Breast Cancer Res. *18*, 21.
13. Loi, S., Michiels, S., Salgado, R., Sirtaine, N., Jose, V., Fumagalli, D., Kellokumpu-Lehtinen, P.L., Bono, P., Kataja, V., Desmedt, C., et al. (2014). Tumor infiltrating lymphocytes are prognostic in triple negative breast cancer and predictive for trastuzumab benefit in early breast cancer: results from the FinHER trial. Ann. Oncol. *25*, 1544–1550.
14. West, N.R., Milne, K., Truong, P.T., Macpherson, N., Nelson, B.H., and Watson, P.H. (2011). Tumor-infiltrating lymphocytes predict response to anthracycline-based chemotherapy in estrogen receptor-negative breast cancer. Breast Cancer Res. *13*, R126.
15. Ali, H.R., Chlon, L., Pharoah, P.D.P., Markowetz, F., and Caldas, C. (2016). Patterns of Immune Infiltration in Breast Cancer and Their Clinical Implications: A Gene-Expression-Based Retrospective Study. M. Ladanyi, ed. *13*, e1002194.
16. RAA, M., Ellis, I.O., Mahmmod, A.M., Hawkes, E.C., Green, A.R., Rakha, E. A., and Martin, S.G. (2011). Lymphatic and blood vessels in basal and triple-negative breast cancers: characteristics and prognostic significance. Mod. Pathol. *24*, 774–785.
17. Wu, S.Z., Al-Eryani, G., Roden, D.L., Junankar, S., Harvey, K., Andersson, A., Thennavan, A., Wang, C., Torpy, J.R., Bartonicek, N., et al. (2021). A single-cell and spatially resolved atlas of human breast cancers. Nat. Genet. *53*, 1334–1347.
18. Costa, A., Kieffer, Y., Scholer-Dahirel, A., Pelon, F., Bourachot, B., Cardon, M., Sirven, P., Magagna, I., Fuhrmann, L., Bernard, C., et al. (2018). Fibroblast Heterogeneity and Immunosuppressive Environment in Human Breast Cancer. Cancer Cell *33*, 463–479.e10.
19. Luca, B.A., Steen, C.B., Matusiak, M., Azizi, A., Varma, S., Zhu, C., Przybyl, J., Espín-Pérez, A., Diehn, M., Alizadeh, A.A., et al. (2021). Atlas of clinically distinct cell states and ecosystems across human solid tumors. Cell *184*, 5482–5496.e28.
20. Yuan, Y., Failmezger, H., Rueda, O.M., Ali, H.R., Gräf, S., Chin, S.F., Schwarz, R.F., Curtis, C., Dunning, M.J., Bardwell, H., et al. (2012). Quantitative Image Analysis of Cellular Heterogeneity in Breast Tumors Complements Genomic Profiling. Sci. Transl. Med. *4*, 157ra143. https://doi.org/10.1126/scitranslmed.3004330.
21. Tran, K.A., Addala, V., Johnston, R.L., Lovell, D., Bradley, A., Koufariotis, L.T., Wood, S., Wu, S.Z., Roden, D., Al-Eryani, G., et al. (2023). Performance of tumour microenvironment deconvolution methods in breast cancer using single-cell simulated bulk mixtures. Nat. Commun. *14*, 5758.
22. Nygaard, V., Rødland, E.A., and Hovig, E. (2016). Methods that remove batch effects while retaining group differences may lead to exaggerated confidence in downstream analyses. Biostatistics *17*, 29–39.
23. Shang, B., Liu, Y., Jiang, S.j., and Liu, Y. (2015). Prognostic value of tumor-infiltrating FoxP3+ regulatory T cells in cancers: a systematic review and meta-analysis. Sci. Rep. *5*, 15179.
24. Monti, M., Ferrari, G., Gazzurelli, L., Bugatti, M., Facchetti, F., and Vermi, W. (2024). Plasmacytoid dendritic cells at the forefront of anti-cancer immunity: rewiring strategies for tumor microenvironment remodeling. J. Exp. Clin. Cancer Res. *43*, 196.
25. Garcia-Recio, S., Hinoue, T., Wheeler, G.L., Kelly, B.J., Garrido-Castro, A. C., Pascual, T., De Cubas, A.A., Xia, Y., Felsheim, B.M., McClure, M.B., Rajkovic, A., et al. (2022). Multiomics in primary and metastatic breast tumors from the AURORA US network finds microenvironment and epigenetic drivers of metastasis. Nat. Cancer *4*, 128–147. https://doi.org/10.1038/s43018-022-00491-x. https://www.nature.com/articles/s43018-022-00491-x.
26. Mao, X., Xu, J., Wang, W., Liang, C., Hua, J., Liu, J., Zhang, B., Meng, Q., Yu, X., and Shi, S. (2021). Crosstalk between cancer-associated fibroblasts and immune cells in the tumor microenvironment: new findings and future perspectives. Mol. Cancer *20*, 131.
27. Hu, M., and Chikina, M. (2024). InstaPrism: an R package for fast implementation of BayesPrism. M. Nikolski, ed. *40*, btae440.
28. Menden, K., Marouf, M., Oller, S., Dalmia, A., Magruder, D.S., Kloiber, K., Heutink, P., and Bonn, S. (2020). Deep learning–based cell composition analysis from tissue expression profiles. Sci. Adv. *6*, eaba2619.
29. Zaitsev, A., Chelushkin, M., Dyikanov, D., Cheremushkin, I., Shpak, B., Nomie, K., Zyrin, V., Nuzhdina, E., Lozinsky, Y., Zotova, A., et al. (2022). Precise reconstruction of the TME using bulk RNA-seq and a machine learning algorithm trained on artificial transcriptomes. Cancer Cell *40*, 879–894.e16.
30. Newman, A.M., Liu, C.L., Green, M.R., Gentles, A.J., Feng, W., Xu, Y., Hoang, C.D., Diehn, M., and Alizadeh, A.A. (2015). Robust enumeration of cell subsets from tissue expression profiles. Nat. Methods *12*, 453–457.
31. Sturm, G., Finotello, F., and List, M. (2020). Immunedeconv: An R Package for Unified Access to Computational Methods for Estimating Immune Cell Fractions from Bulk RNA-Sequencing Data. In Bioinformatics for Cancer Immunotherapy, S. Boegel, ed. (Springer US), pp. 223–232. http://link.springer.com/10.1007/978-1-0716-0327-7_16.
32. Van Loo, P., Nordgard, S.H., Lingjærde, O.C., Russnes, H.G., Rye, I.H., Sun, W., Weigman, V.J., Marynen, P., Zetterberg, A., Naume, B., Perou, C.M., et al. (2010). Allele-specific copy number analysis of tumors. Proc. Natl. Acad. Sci. USA *107*, 16910–16915.
33. Barreca, M., Dugo, M., Galbardi, B., Győrffy, B., NA-PHER2 consortium; NeoTRIP consortium; Valagussa, P., Besozzi, D., Viale, G., Bianchini, G., Gianni, L., et al. (2024). Development and validation of a gene expression score to account for tumour purity and improve prognostication in breast cancer. Preprint at bioRxiv. https://doi.org/10.1101/2024.02.23.581701.
34. Danenberg, E., Bardwell, H., Zanotelli, V.R.T., Provenzano, E., Chin, S.F., Rueda, O.M., Green, A., Rakha, E., Aparicio, S., Ellis, I.O., et al. (2022). Breast tumor microenvironment structures are associated with genomic features and clinical outcome. Nat. Genet. *54*, 660–669.
35. Finotello, F., Mayer, C., Plattner, C., Laschober, G., Rieder, D., Hackl, H., Krogsdam, A., Loncova, Z., Posch, W., Wilflingseder, D., et al. (2019). Molecular and pharmacological modulators of the tumor immune contexture revealed by deconvolution of RNA-seq data. Genome Med. *11*, 34.
36. Becht, E., Giraldo, N.A., Lacroix, L., Buttard, B., Elarouci, N., Petitprez, F., Selves, J., Laurent-Puig, P., Sautès-Fridman, C., Fridman, W.H., and de Reyniès, A. (2016). Estimating the population abundance of tissue-infiltrating immune and stromal cell populations using gene expression. Genome Biol. *17*, 218.

CellPress
OPEN ACCESS

37. Aran, D., Hu, Z., and Butte, A.J. (2017). xCell: digitally portraying the tissue cellular heterogeneity landscape. Genome Biol. *18*, 220.
38. Racle, J., De Jonge, K., Baumgaertner, P., Speiser, D.E., and Gfeller, D. (2017). Simultaneous enumeration of cancer and immune cell types from bulk tumor gene expression data. eLife *6*, e26476.
39. Yoshihara, K., Shahmoradgoli, M., Martínez, E., Vegesna, R., Kim, H., Torres-Garcia, W., Treviño, V., Shen, H., Laird, P.W., Levine, D.A., et al. (2013). Inferring tumour purity and stromal and immune cell admixture from expression data. Nat. Commun. *4*, 2612.
40. Monaco, G., Lee, B., Xu, W., Mustafah, S., Hwang, Y.Y., Carré, C., Burdin, N., Visan, L., Ceccarelli, M., Poidinger, M., et al. (2019). RNA-Seq Signatures Normalized by mRNA Abundance Allow Absolute Deconvolution of Human Immune Cell Types. Cell Rep. *26*, 1627–1640.e7.
41. Jiménez-Sánchez, A., Cast, O., and Miller, M.L. (2019). Comprehensive Benchmarking and Integration of Tumor Microenvironment Cell Estimation Methods. Cancer Res. *79*, 6238–6246.
42. Chu, T., Wang, Z., Pe'er, D., and Danko, C.G. (2022). Cell type and gene expression deconvolution with BayesPrism enables Bayesian integrative analysis across bulk and single-cell RNA sequencing in oncology. Nat. Cancer *3*, 505–517.
43. Batra, R.N., Lifshitz, A., Vidakovic, A.T., Chin, S.F., Sati-Batra, A., Sammut, S.J., Provenzano, E., Ali, H.R., Dariush, A., Bruna, A., et al. (2021). DNA methylation landscapes of 1538 breast cancers reveal a replication-linked clock, epigenomic instability and cis-regulation. Nat. Commun. *12*, 5406.
44. Cook, S. (2019). Forecast Evaluation Using Theil's Inequality Coefficients (The Economics Network). https://www.economicsnetwork.ac.uk/showcase/cook_theil.
45. Ali, H.R., Rueda, O.M., Chin, S.F., Curtis, C., Dunning, M.J., Aparicio, S.A., and Caldas, C. (2014). Genome-driven integrated classification of breast cancer validated in over 7,500 samples. Genome Biol. *15*, 431.
46. Gendoo, D.M.A., Ratanasirigulchai, N., Schröder, M.S., Paré, L., Parker, J.S., Prat, A., and Haibe-Kains, B. (2016). Genefu: an R/Bioconductor package for computation of gene expression-based signatures in breast cancer. Bioinformatics *32*, 1097–1099.
47. Thennavan, A., Beca, F., Xia, Y., Recio, S.G., Allison, K., Collins, L.C., Tse, G.M., Chen, Y.Y., Schnitt, S.J., Hoadley, K.A., et al. (2021). Molecular analysis of TCGA breast cancer histologic types. Cell Genom. *1*, 100067.
48. Scrucca, L., Fop, M., Murphy, T.B., and Raftery, A.E. (2016). mclust 5: Clustering, Classification and Density Estimation Using Gaussian Finite Mixture Models. R J. *8*, 289–317.
49. Van Den Boogaart, K.G., and Tolosana-Delgado, R. (2008). "compositions": A unified R package to analyze compositional data. Comput. Geosci. *34*, 320–338.

STAR★METHODS

KEY RESOURCES TABLE

REAGENT or RESOURCE	SOURCE	IDENTIFIER
Deposited data		
METABRIC Expression + Clinical Data	METABRIC	https://www.cbioportal.org/study/summary?id=brca_metabric
TCGA Expression + Clinical Data	TCGA	https://www.cbioportal.org/study/summary?id=brca_tcga
MBC Expression + Clinical Data	The Metastatic Breast Cancer Project	https://www.cbioportal.org/study/summary?id=brca_mbcproject_2022
SCAN-B Expression + Clinical Data	Swedish Cancerome Analysis Network - Breast	https://doi.org/10.17632/yzxtxn4nmd.3
SMC Expression + Clinical Data	Samsung Medical Center Korean Breast Cancer cohort	https://www.cbioportal.org/study/summary?id=brca_smc_2018
I-SPY2 Expression + Clinical Data	I-SPY 2 Clinical Trial	NCBI GEO: GSE194040
Hatiz-Validation Expression + Clinical Data	Nwosu 2023	https://osf.io/eky3p/: GSE25065
Hatiz-Discovery Expression + Clinical Data	Nwosu 2023	https://osf.io/eky3p/: GSE25055
AURORA Expression + Clinical Data	AURORA US Metastasis Project	NCBI GEO: GSE209998
TransNEO Expression + Clinical Data	Sammut, 2022	https://github.com/cclab-brca/neoadjuvant-therapy-response-predictor
MyBrca Expression + Clinical Data	Soo-Hwang Teo	Data was requested directly from the MyBrCa Tumor Genomics Data Access Committee
Derouane Expression + Clinical Data	Derouane 2024	NCBI GEO: GSE240671
CALGB Expression + Clinical Data	CALGB Clinical Trial	NCBI GEO: GSE154524
Matador Expression + Clinical Data	MATADOR Clinical Trial	NCBI GEO: GSE167977
IMC Data	Danenberg 2022	https://zenodo.org/records/5850952
Methylayer	Batra 2021	https://github.com/tanaylab/metabric_rrbs
Digital Pathology	Yuan 2012	Raw data was requested directly from the authors. Access should be requested directly from the data maintainers.
Software and algorithms		
R (v4.3.2)	R Core Team	RRID:SCR_001905
InstaPrism	Hu 2024[27]	https://github.com/humengying0907/InstaPrism
Scaden	Menden 2020[28]	https://github.com/KevinMenden/scaden
Kassandra	Zaitsev 2022[29]	https://science.bostongene.com/kassandra/
Cibersort	Newman 2015[30]	https://cibersortx.stanford.edu/
Immunedeconv	Sturm 2020[31]	https://github.com/omnideconv/immunedeconv
ASCAT	Van Loo 2010[32]	https://github.com/VanLoo-lab/ascat
BCPS	Barreca 2024[33]	https://github.com/BarrecaMarco/BCPS
Code	This paper	https://doi.org/10.17605/OSF.IO/B8VYT

EXPERIMENTAL MODEL AND STUDY PARTICIPANT DETAILS

The study protocol received approval from the institutional review boards or ethics committees of the participating institutions. As this study is retrospective, patient consent was not required.

In the benchmarking portion of this study, IMC data was used as the ground truth for immune populations within the 693 samples from the METABRIC cohort and downloaded from.[34] Briefly, IMC data was collected from FFPE treatment-naive primary BC tissue. Suitable areas of cancer sections were selected by a pathologist and punched using a manual microarrayer (0.6–1.0 mm diameter). A total of

749 IMC images corresponding to 693 patients (635 tumors were represented by one tissue spot, 55 by two and 3 by three). Tissue level and cell-level TME features were matched as described in Table S5. This tissue-level analysis was intended to provide a comparison between TME characterization methods at coarser resolutions (i.e., stroma, immune, and epithelial/tumor compartments).

In the meta-analysis portion of this study, 16,337 primary breast cancer patients from thirteen different studies. The demographic characteristics of the cohort are detailed in Table S2. Given the multi-institutional nature of the dataset, the gene expression profiling methods panels used varied. Three of the studies used microarray panels (n = 3,444) and ten studies used RNAseq (n = 12,947). The original 16,337 samples were corrected to 14,837 after removing samples in which deconvolution failed (0 was returned for all cell types) or duplicate patients were identified (Figure 2). Additional details on panel specifications are provided in Table S1.

METHOD DETAILS

TME characterization

The list of deconvolution methods can be found in Table S4 and the expression files used from each study can be found in Table S1. The immunedeconv R package was used to access Quantiseq, TIMER, MCPCounter, xCell, EPIC, ESTIMATE, ABIS, and ConsensusTME as well as the training data that accompanied each method.[30,31,35–41] Because TIMER and ConsensusTME use indication-specific reference profiles, the ’brca’ tumor type was specified. CIBERSORT was accessed through its maintainer’s web portal (https://cibersortx.stanford.edu/).[30] The LM22 signature matrix file was run at 1,000 permutations. InstaPrism is a fast implementation of the BayesPrism deconvolution algorithm.[27,42] The scRNAseq training file was created as per the standard files available with the packages using the BC single cell atlas from.[17] Scaden was installed as per the authors instructions through Bioconda and trained on human PBMC data provided by the maintainers.[28] BCPS was run as instructed by the maintainer.[33] Kassandra was accessed through the maintainer’s web portal (https://science.bostongene.com/kassandra).[29] Digital pathology data was accessed from.[20] Methylayer data, which is a reference-free method, was accessed from.[43] ASCAT scores, also determined using a reference-free approach, were based on copy number and B allele frequencies (allelic imbalance) data from germline SNPs.[32] Data was omitted in cases where the deconvolution methods failed (defined as when a method returned an ’NA’ value for a given sample).

Benchmarking of characterization methods

Image mass cytometry (IMC) is an imaging technique that allows for the simultaneous detection and quantification of up to 40 biomarkers at the single-cell level within tissue samples. IMC data was used as the ground truth for immune populations within the 693 samples from the METABRIC cohort and downloaded from.[34] A total of 749 IMC images corresponding to 693 patients (635 tumors were represented by one tissue spot, 55 by two and 3 by three). Tissue level and cell-level TME features were matched as described in Table S5. This tissue-level analysis was intended to provide a comparison between TME characterization methods at coarser resolutions (i.e., stroma, immune, and epithelial/tumor compartments). The proportion of each immune cell was calculated by finding the total number of cells as represented in the IMC data for each sample, then dividing the count of each cell by the total. Abundance scores from deconvolution were transformed to be on a 0–1 scale with scores adding up to 1. Median absolute error (MDAE) between samples with matched IMC-deconvolution derived cell type fractions was determined *a priori* to be the primary benchmarking score and determined using the DescTools package.[44] Root mean squared error (RMSE) was used as the secondary benchmarking score. Cellularity was determined preferentially by pathologist-based annotation in the original METABRIC study from which the patient data was derived; if this data was not available, it was determined via IMC, where epithelial fraction was split into tertiles for ’low’, ’moderate’, and ’high’ cellularity respectively.

Collation of *meta*-dataset

Thirteen BC studies meeting eligibility criteria—gene expression profiles and clinical data from ≥100 primary tumors per study—were included (Table S1). Datasets were accessed in February 2024. Samples failing deconvolution or exhibiting duplicate patient identifiers within the same study were excluded to mitigate technical and biological redundancy (Figure 2A). Clinical and molecular annotations were preferentially derived from study-provided metadata; where unavailable, IntClust subtypes were classified using gene expression data via the ic10 R package,[45] and PAM50 subtypes were assigned using the genefu R package.[46] ER and HER2 status for TCGA samples were annotated as per prior studies[47]; remaining cases utilized the MClust package for classification.[48] Following *meta*-dataset assembly, deconvolution was performed using InstaPrism, prioritizing raw sequencing data over transcripts per million normalized values where available.

In total, 13 studies were included and the source of the data for each data are described in Table S1. All datasets were downloaded in February 2024. All BC studies with gene expression data and clinical data from at >100 primary breast tumors were considered eligible. Primary tumor samples that were unable to be deconvoluted or those that had the same patient identifier in the same study were removed (Figure 2A). Clinical and molecular annotation was preferentially determined by study annotation if this data was available. The classification into the ten IntClust subtypes was achieved based on gene expression data using the ic10 package in R, as previously described.[45] Classification into the PAM50 subtypes were completed using the genefu R package.[46] ER and HER2 status annotation in the TCGA dataset was determined as from[47]; otherwise, ER and HER2 status were determined using the MClust package.[48] After the *meta*-dataset was collated, samples were run through InstaPrism; raw data, if available, was preferentially used for deconvolution instead of transcripts per million data. A glossary of the InstaPrism cell types is available in Table S6.

Identification of microenvironment signatures

To minimize confounding inter-study variability, we restricted microenvironmental signature analysis to the SCAN-B cohort, the largest dataset in our meta-analysis. We designated the resulting clusters "TME Types" to distinguish them from previously published "ecotypes," acknowledging that our approach differs in the underlying patient cohort, data modality (RNA-seq), and deconvolution algorithm used. Compositional cell-subtype data were transformed using the Aitchison centered log-ratio transformation to account for data sparsity and compositional bias using the compositions R package.[49] Transformed values were standardized to Z-scores for each subtype, and k-means clustering (Euclidean distance, 1,000 maximum iterations) was performed using Morpheus. The optimal cluster number was determined via Bayesian Information Criterion (BIC)-guided expectation maximization, implemented through hierarchical clustering-initialized Gaussian mixture models in the *MClust* package.[48] PAM50 subtype enrichment within clusters was assessed using chi-square tests, and BCSS was evaluated for each TME signature, stratified by PAM50 subtype using Kaplan Meier curves.

Assessing disease-specific, relapse-free, and metastasis-free survival

Multivariable Cox proportional hazards models were employed to assess BCSS, RFS, and MFS. Models included TME cell subtypes (quartilized to mitigate sparse cell population biases) as primary variables of interest, adjusted for tumor stage, grade, nodal involvement, and study-specific variability (incorporated as strata). Analyses were conducted separately for ER+ and ER-cohorts using the survival R package. For RFS, time-stratified log rank tests were implemented to evaluate period-specific relapse risks: short-term relapse (≤10 years) was analyzed using follow-up data censored at 10 years, while long-term relapse (>10 years) utilized left-truncated survival data restricted to patients' event-free beyond this threshold. Cell subtypes for long-term relapse interrogation were systematically selected based on significant log rank effects ($p < 0.05$) identified in the post-10-year survival analysis, stratified by ER status.

Treatment response

To study treatment response, we used a generalized linear model to calculate odds ratio for pathological complete response. The regression was controlled with treatment type, study, and age.[9] We conducted separate analyses for ER- and ER + BC, and for chemotherapy and immunotherapy/immunotherapy + chemotherapy combination therapy. For immunotherapy, we used consolidated cell types into parent categories due to a smaller sample size. After identifying the most significant cell types that promote and prevent treatment response, we Systematically evaluated for trends of quartilized populations of cell subtypes in their families with RCB treatment response. Spine plots to highlight were selected after systematically evaluating each cell subtype for trends.

To evaluate associations between TME composition and chemotherapeutic efficacy, we performed logistic regression using pathological complete response as the binary outcome, adjusted for treatment type, study cohort, and patient age.[9] Analyses were stratified by ER status. We conducted the same analysis for patients treated with immunotherapy or immunotherapy-chemotherapy combinations. For immunotherapy cohorts, sparse cell subtype populations necessitated aggregation into broader immune lineage categories (e.g., "T cells" vs. $CD8^+$/$CD4^+$ subsets). Furthermore, due to a limited sample size, we were only able to study the ER + subtype. Cell subtypes significantly associated with pathological complete response were analyzed for dose-response trends by quartilizing their abundances, with residual cancer burden outcomes evaluated for both the most strongly positively and negatively associated lineage categories. Visualization of these relationships was prioritized using spine plots, which were selectively generated for subtypes exhibiting monotonic or threshold-dependent trends across quartiles.

Metastatic composition

To assess TME heterogeneity between metastatic and primary BC tumors, we analyzed deconvoluted transcriptional profiles from paired samples.[25] Paired t-tests were performed for metastatic sites with ≥3 matched primary-metastasis pairs (brain, chest wall, lung, and liver). Cell subtype abundances were compared to primary tumor counterparts, with log2-fold changes quantified to evaluate compositional shifts. Cases showing apparent subtype switching (e.g., luminal-to-basal transition) were initially excluded; however, as no significant differences in cancer cell subtype distributions emerged between switched and non-switched cohorts, all cases were retained to ensure analytical inclusivity.

QUANTIFICATION AND STATISTICAL ANALYSIS

All statistical analysis was conducted using R (v4.3.2). Centered log-ratio transformations were performed using compositions (v2.0-8). Survival analyses were conducted using the R packages survival (version 3.5.7) and survminer (version 0.4.9). *p*-values <0.05 and 95% CI were considered significant. If applicable, *p*-values were adjusted using Benjamini-Hochberg correction. Cancer fractions from tumor composition were removed to only consider TME features, and the proportion of each feature was considered as a fraction of the noncancerous TME. The optimal number of TME Types was determined via Bayesian Information Criterion (BIC)-guided expectation maximization, implemented through hierarchical clustering-initialized Gaussian mixture models in the MClust package (version 6.11).

Cell Reports Medicine, Volume *6*

Supplemental information

The tumor microenvironment of 14,837 breast cancers is associated with clinical outcome independently of genomic subtypes

Kevin J. Tu, Daniel Guerrero-Romero, Kate Eason, Raquel Manzano Garcia, Jia Wern, Soo-Hwang Teo, Long Nguyen, Stephen-John Sammut, Florian Markowetz, Oscar M. Rueda, and Carlos Caldas

Supplementary Figures

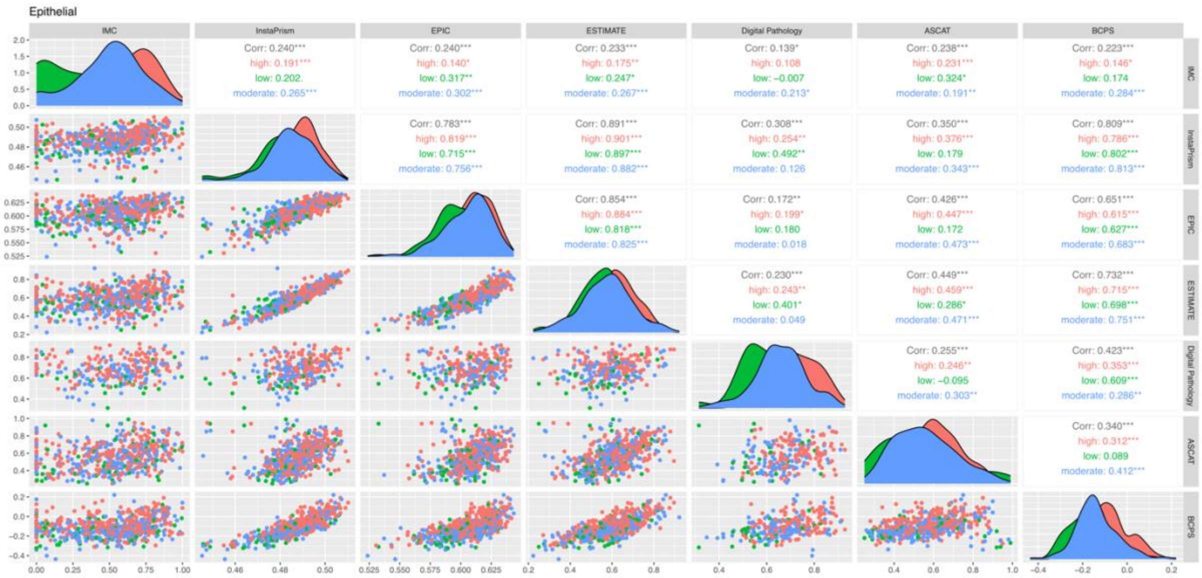

Supplementary Figure 1. Scatterplot matrix of epithelial characterization. Red is high cellularity samples, green is low cellularity samples, and blue is moderate cellularity samples according to pathologist's assessment. * $p < 0.05$, ** $p < 0.01$, *** $p < 0.001$, **** $p < 0.0001$. Related to Figure 1.

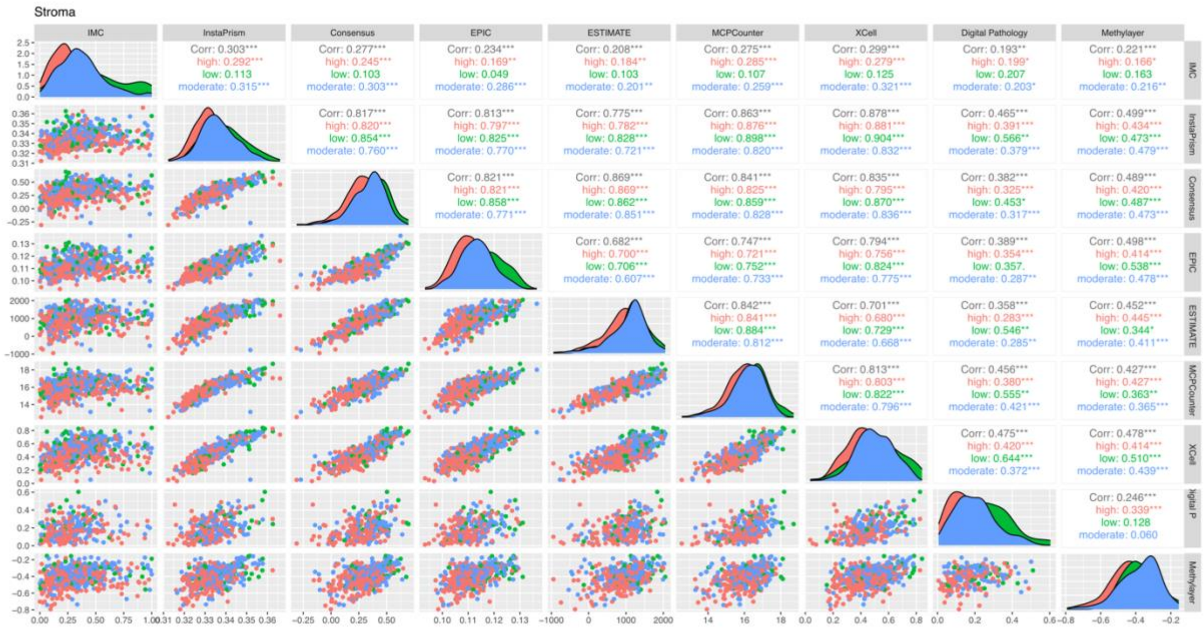

Supplementary Figure 2. Scatterplot matrix of stroma characterization. Red is high cellularity samples, green is low cellularity samples, and blue is moderate cellularity samples. * $p < 0.05$, ** $p < 0.01$, *** $p < 0.001$, **** $p < 0.0001$. Related to Figure 1.

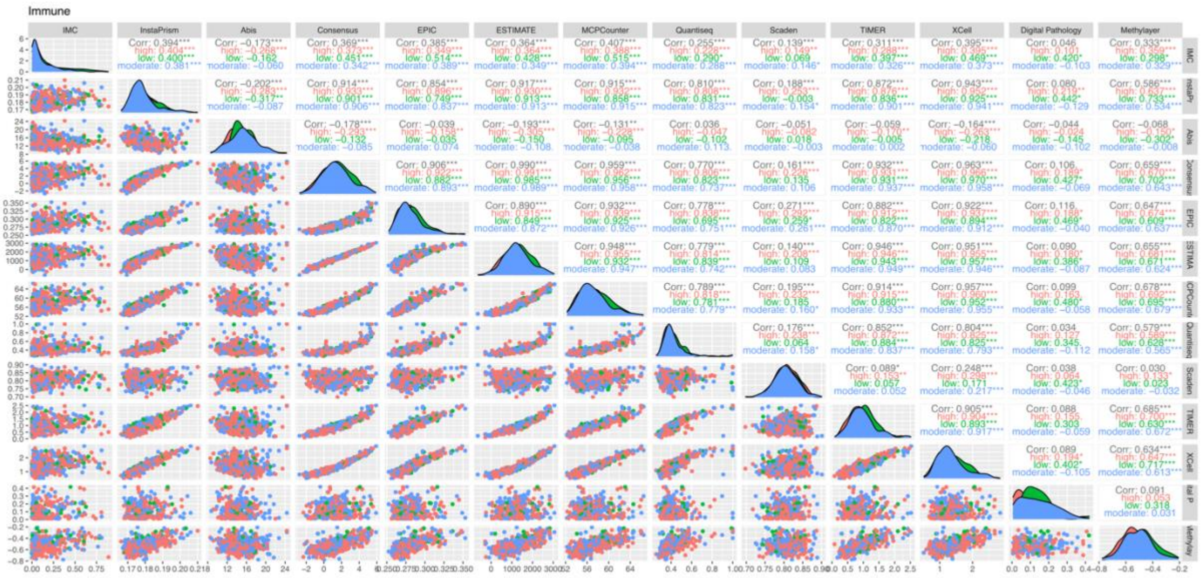

Supplementary Figure 3. Scatterplot matrix of immune characterization. Red is high cellularity samples, green is low cellularity samples, and blue is moderate cellularity samples. * p < 0.05, ** p < 0.01, *** p < 0.001, **** p < 0.0001. Related to Figure 1.

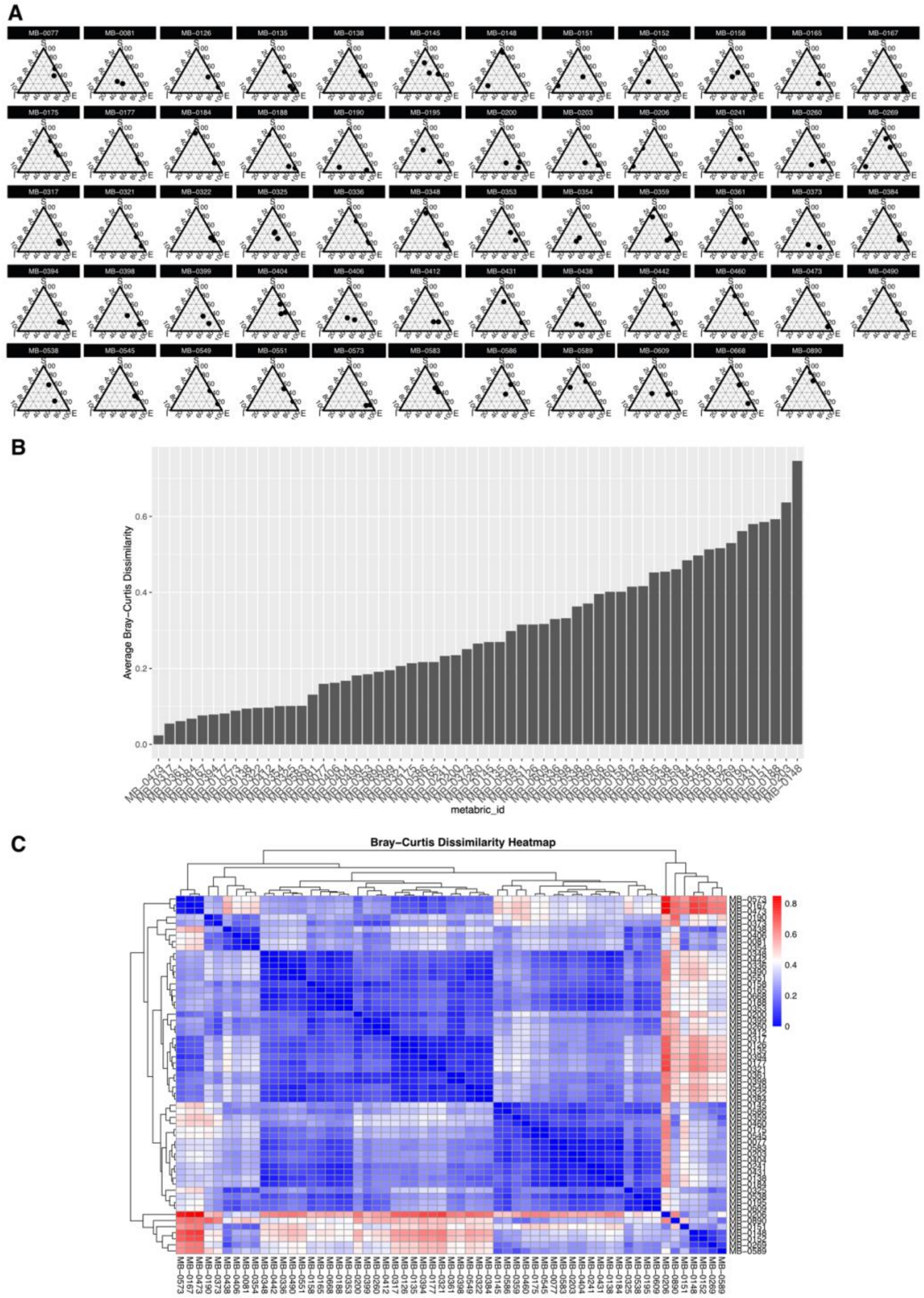

Supplementary Figure 4. IMC Sample Variation. A) Ternary plots representing the stroma (S), immune (I), and epithelial (E) fractions of patients with multiple IMC samples. B) Average pairwise Bray-Curtis dissimilarity between sample makeup for each tumor. C) Dissimilarity matrix of the average pairwise Bray-Curtis dissimilarity between tumors, clustering by Euclidean distance. Related to Figure 1.

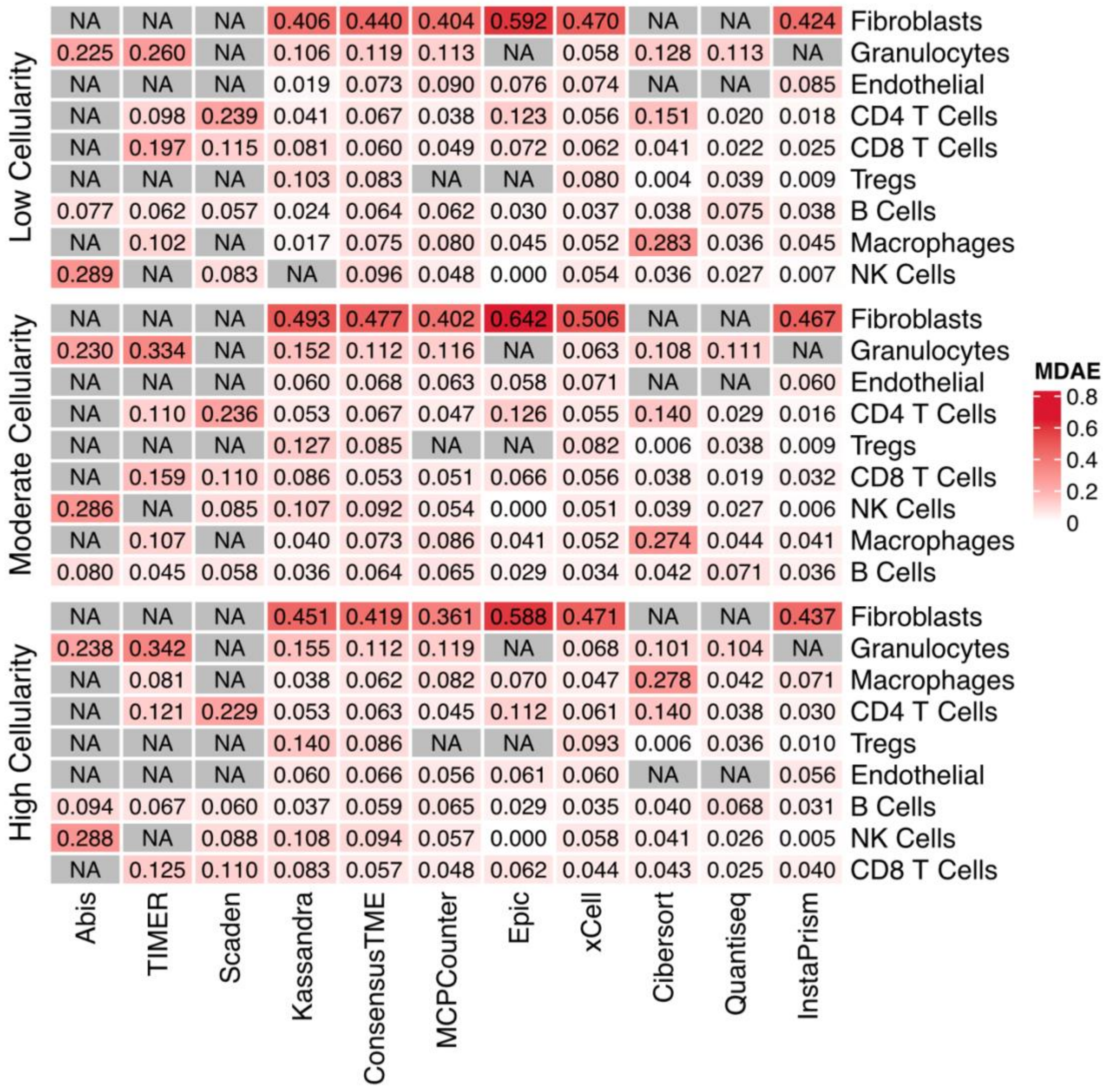

Supplementary Figure 5. Deconvolution algorithm benchmarking using MDAE. Samples are ordered from left to right and top to bottom by decreasing median MDAE. Related to Figure 1.

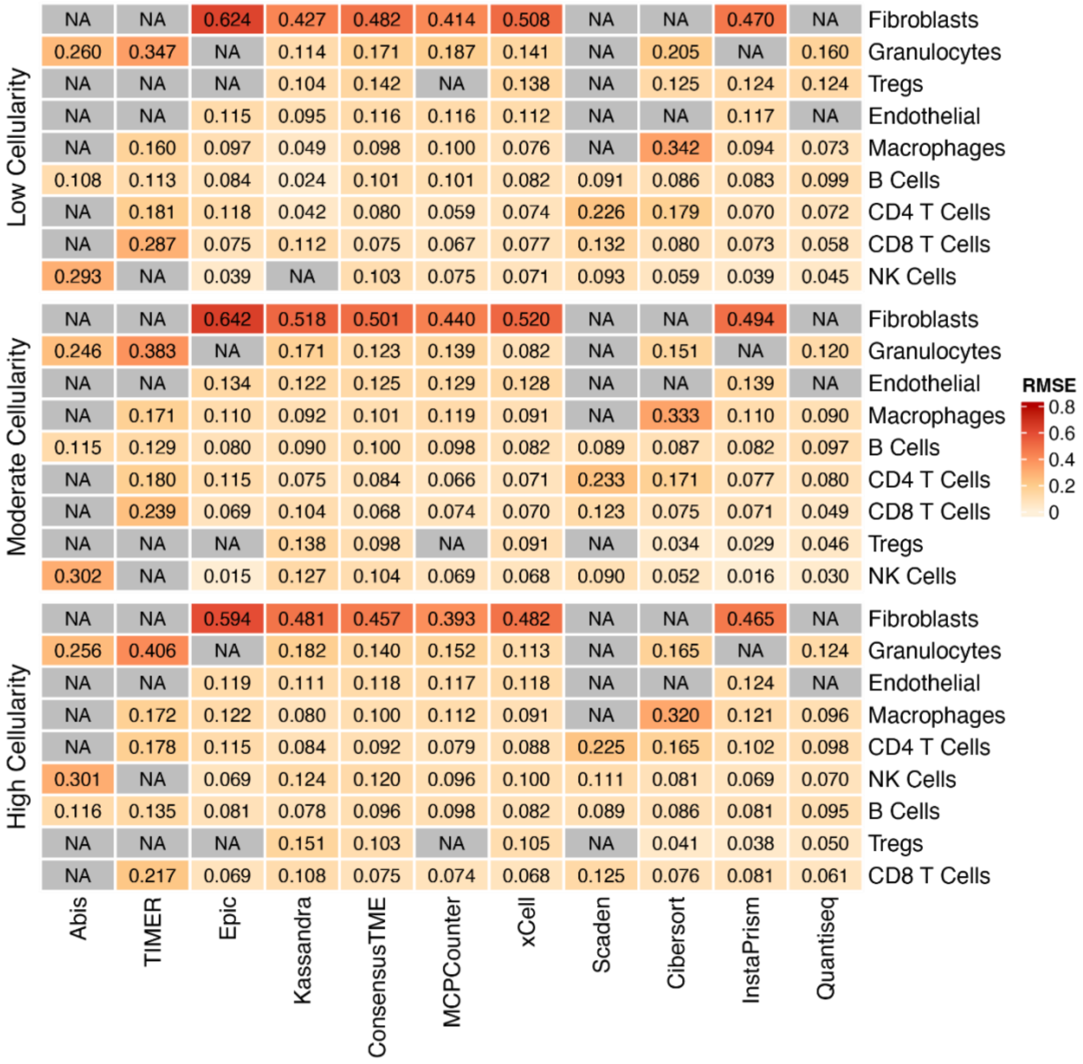

Supplementary Figure 6. Deconvolution algorithm benchmarking using RMSE. Samples are ordered from left to right and top to bottom by decreasing median RMSE. Related to Figure 1.

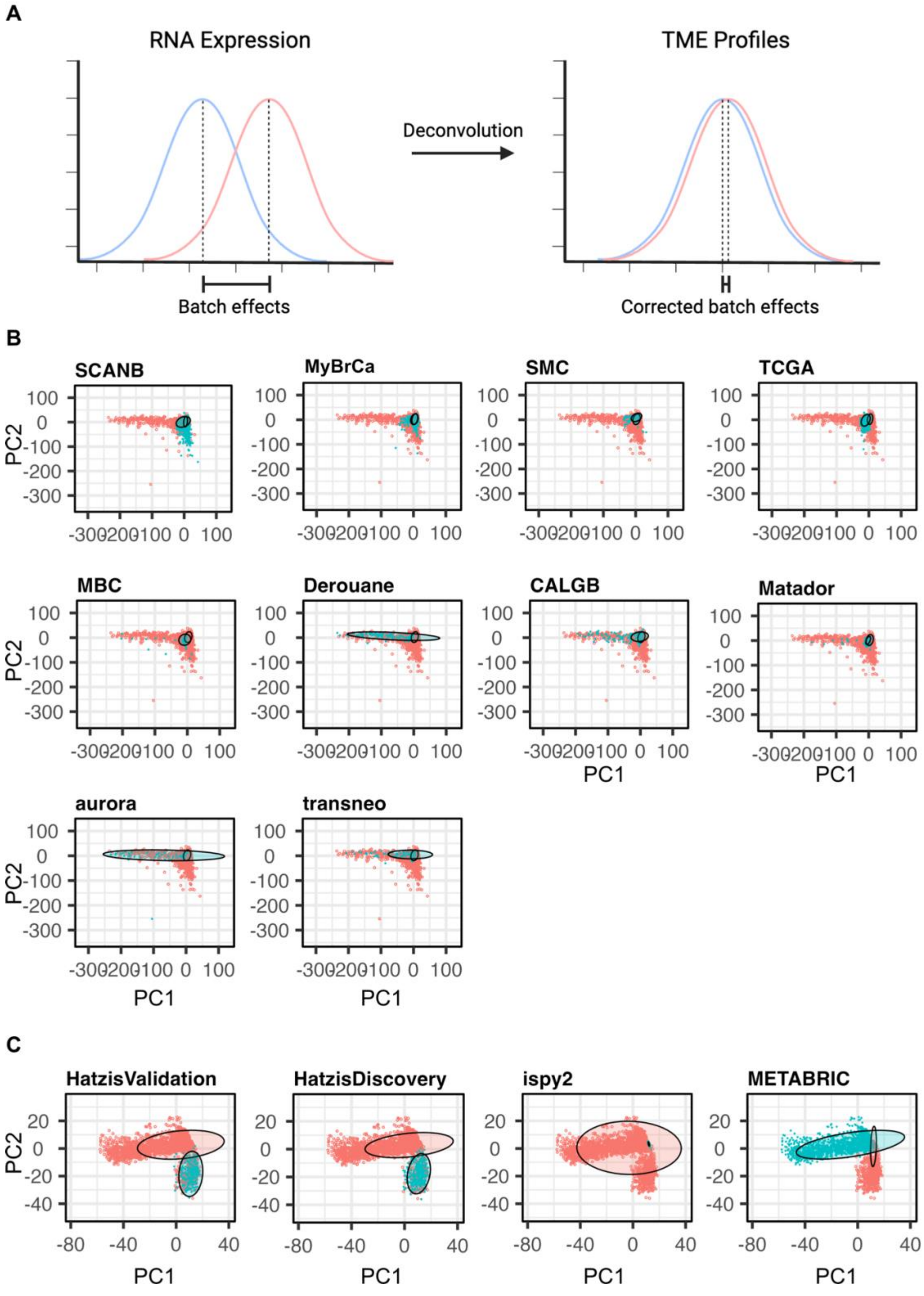

Supplementary Figure 7. Correction of batch effects by deconvolution.

A) Fictional example of translation of TME proportions to account for batch effects through deconvolution. B) PCA plots colored by RNAseq study, the study is in blue and all other studies are in red. C) PCA plots colored by microarray study, the study is in blue and all other studies are in red. Related to Figure 2.

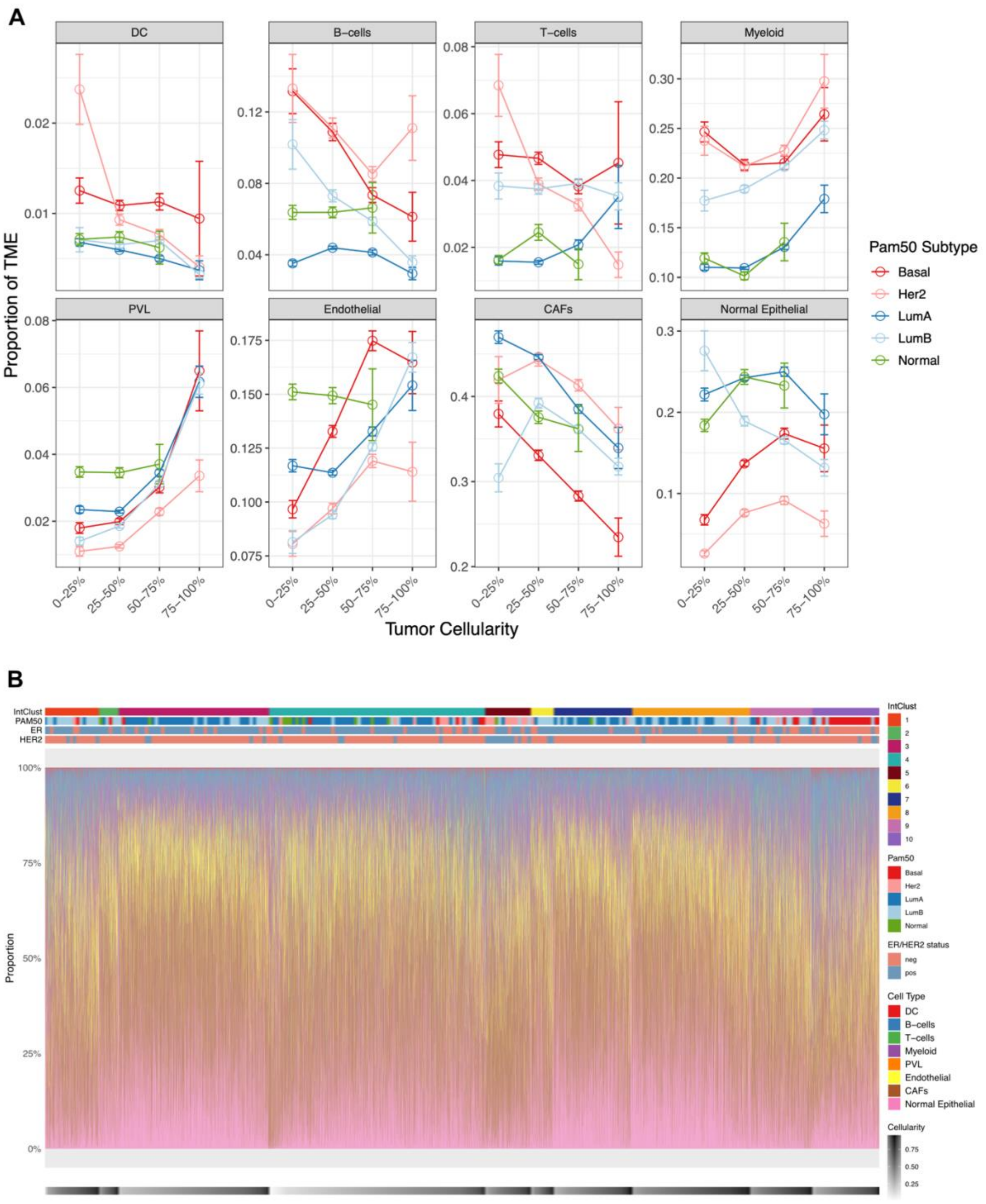

Supplementary Figure 8. TME profiles in Pam50 and IntClust subtypes. A) Comparing cell types between the instrinsic subtypes over cellularity. B) The TME profiles of all patients in our meta-dataset grouped by integrative cluster. Related to Figure 3.

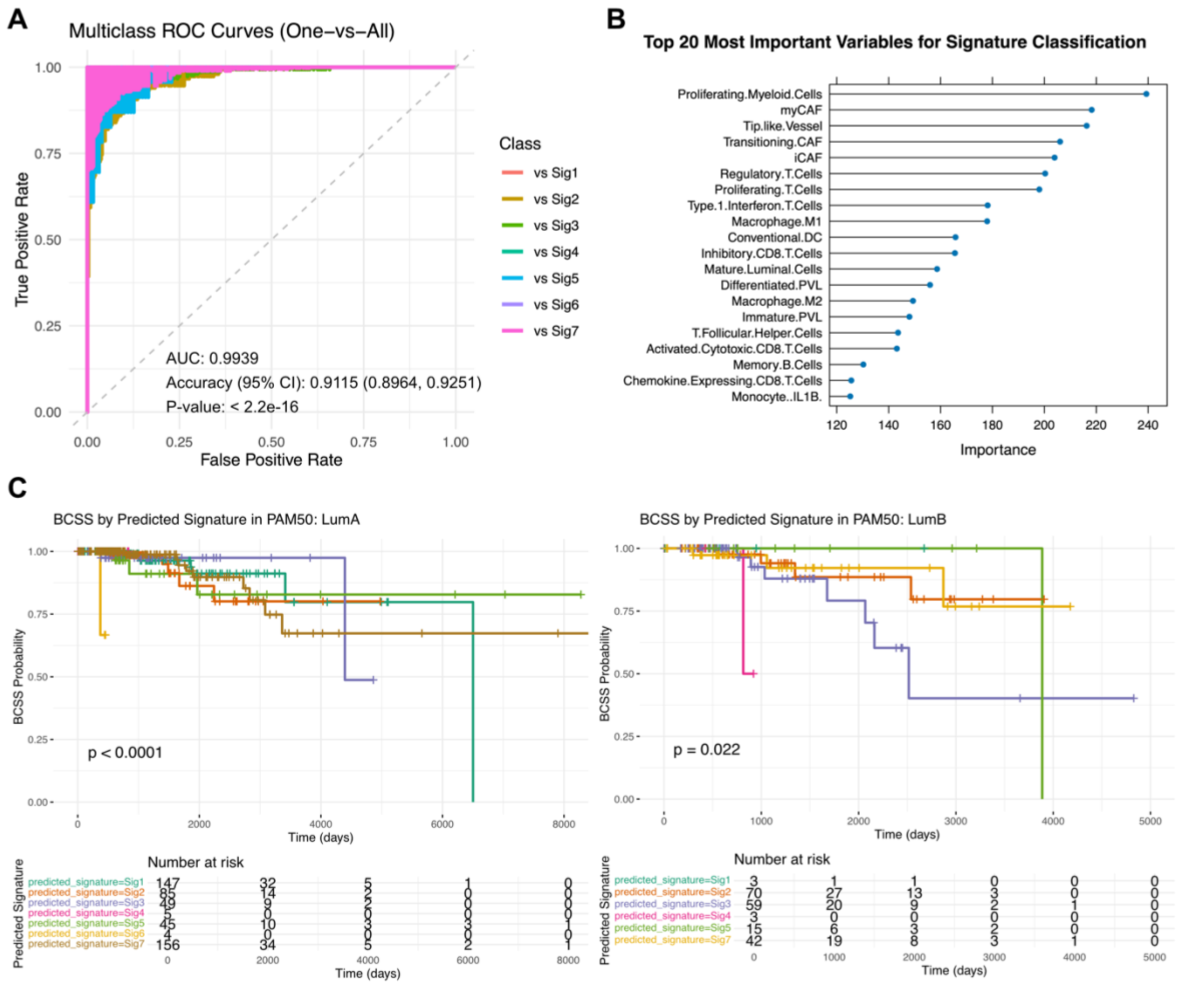

Supplementary Figure 9. Validation of Ecotypes within the TCGA cohort. A) AUC curve of random forest classifier. B) Confusion matrix of random forest classifier. C) Variable importance for the classifier. D) BCSS KM curves of TME types within Luminal subtypes. Related to Figure 4.

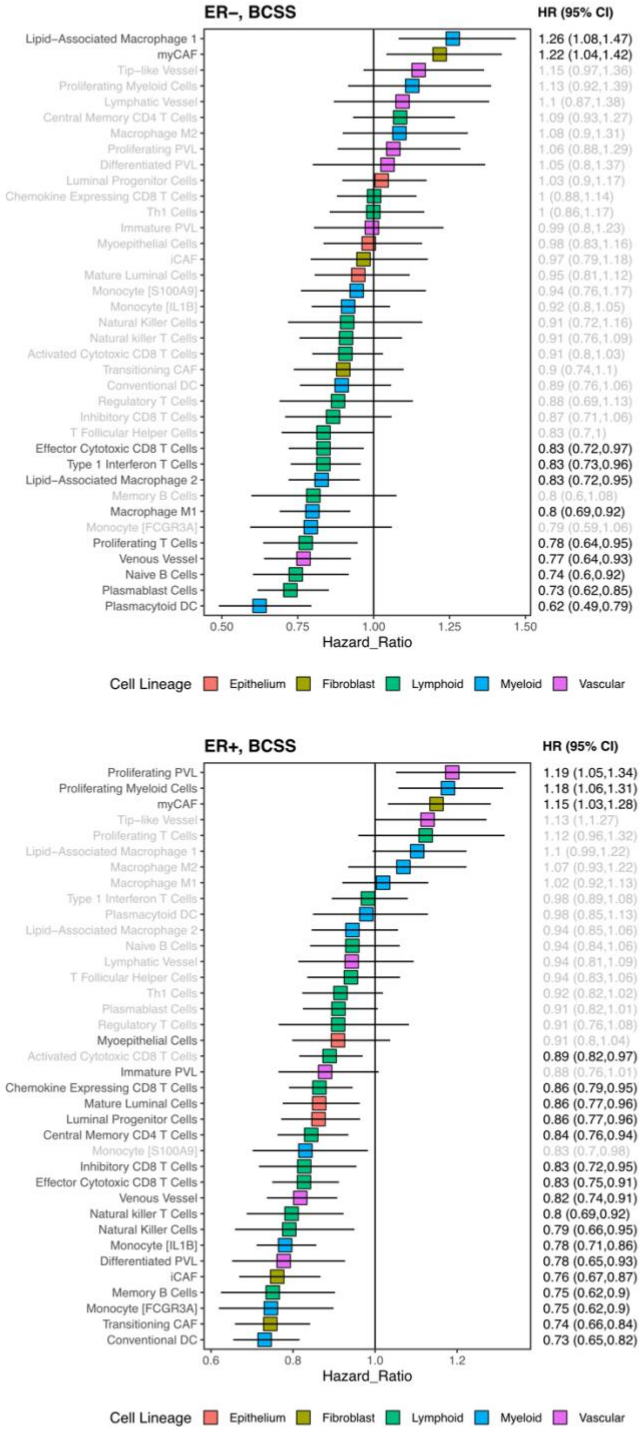

Supplementary Figure 10. The effects of TME features on disease-specific survival. A) Adjusted Cox model in ER+ patients for BCSS. B) Adjusted Cox model in ER- patients for BCSS. Related to Figure 5.
